# Effects of ketamine on brain function during metacognition of episodic memory

**DOI:** 10.1093/nc/niaa028

**Published:** 2021-02-10

**Authors:** Mirko Lehmann, Claudia Neumann, Sven Wasserthal, Johannes Schultz, Achilles Delis, Peter Trautner, René Hurlemann, Ulrich Ettinger

**Affiliations:** Department of Psychology, University of Bonn, Bonn, Germany; Department of Anesthesiology and Intensive Care Medicine, University Hospital Bonn, Bonn, Germany; Department of Psychiatry and Division of Medical Psychology, University Hospital Bonn, Bonn, Germany; Center for Economics and Neuroscience, University of Bonn, Bonn, Germany; Institute for Experimental Epileptology and Cognition Research, University of Bonn Medical Center, Bonn, Germany; Department of Anesthesiology and Intensive Care Medicine, University Hospital Bonn, Bonn, Germany; Center for Economics and Neuroscience, University of Bonn, Bonn, Germany; Institute for Experimental Epileptology and Cognition Research, University of Bonn Medical Center, Bonn, Germany; Department for NeuroCognition, Life & Brain Center, Bonn, Germany; Department of Psychiatry and Division of Medical Psychology, University Hospital Bonn, Bonn, Germany; Department of Psychiatry, School of Medicine & Health Sciences, University of Oldenburg, Oldenburg, Germany; Research Center Neurosensory Science, University of Oldenburg, Oldenburg, Germany; Department of Psychology, University of Bonn, Bonn, Germany

**Keywords:** metacognition, confidence, ketamine, episodic memory, glutamate

## Abstract

Only little research has been conducted on the pharmacological underpinnings of metacognition. Here, we tested the modulatory effects of a single intravenous dose (100 ng/ml) of the *N*-methyl-D-aspartate-glutamate-receptor antagonist ketamine, a compound known to induce altered states of consciousness, on metacognition and its neural correlates. Fifty-three young, healthy adults completed two study phases of an episodic memory task involving both encoding and retrieval in a double-blind, placebo-controlled fMRI study. Trial-by-trial confidence ratings were collected during retrieval. Effects on the subjective state of consciousness were assessed using the 5D-ASC questionnaire. Confirming that the drug elicited a psychedelic state, there were effects of ketamine on all 5D-ASC scales. Acute ketamine administration during retrieval had deleterious effects on metacognitive sensitivity (meta-d′) and led to larger metacognitive bias, with retrieval performance (d′) and reaction times remaining unaffected. However, there was no ketamine effect on metacognitive efficiency (meta-d′/d′). Measures of the BOLD signal revealed that ketamine compared to placebo elicited higher activation of posterior cortical brain areas, including superior and inferior parietal lobe, calcarine gyrus, and lingual gyrus, albeit not specific to metacognitive confidence ratings. Ketamine administered during encoding did not significantly affect performance or brain activation. Overall, our findings suggest that ketamine impacts metacognition, leading to significantly larger metacognitive bias and deterioration of metacognitive sensitivity as well as unspecific activation increases in posterior hot zone areas of the neural correlates of consciousness.

## Introduction

Many of our thoughts in everyday life revolve around other thoughts, about something we said or a decision we made. It has been postulated that these *meta*-thoughts constitute a distinct feature of consciousness. According to Block (1995), consciousness can be divided into phenomenal consciousness, access consciousness, self-consciousness, and monitoring consciousness. The latter concerns metacognition, i.e., the ability to reflect upon our own thoughts and knowledge and to monitor the quality of ongoing cognitive or perceptual processes (Grimaldi *et al.* 2015). The link between metacognition and consciousness is based on the intuition that, if an individual is unable to reflect on a particular mental state, this state cannot be conscious and consequently, some kind of metacognition should accompany all conscious representations (Shea and Frith 2019).

Metacognition is frequently measured on a trial-by-trial-basis as participants indicate their level of confidence about the accuracy of a perceptual or mnestic judgment (Grimaldi *et al.* 2015). A second-order confidence rating (Type 2 response) is therefore based on a first-order judgment (Type 1 response). Measures of metacognitive sensitivity tap how well participants introspectively assess or monitor their own cognitive processes (Fleming and Lau 2014). By applying signal-detection-theory (SDT) methodology, metacognitive *sensitivity* (as meta-d′) can be quantified independently of interindividual differences in response tendencies (Maniscalco and Lau 2012). The meta-d′-framework also allows to control for the influence of primary task performance on metacognitive sensitivity (Maniscalco and Lau 2014): metacognitive *efficiency* (meta-d′/d′) represents the amount of signal strength available for the metacognitive process, expressed as a fraction of the amount of signal strength available for the Type 1 task (McCurdy *et al.* 2013). Finally, it is important to consider the general tendency for higher or lower confidence ratings, the so-called metacognitive *bias* (Fleming and Lau 2014).

But what is the neural basis of metacognition? By drawing on evidence from no-report paradigms, Koch *et al.* (2016) argue that the neural correlates of consciousness are primarily localized in a posterior cortical network labeled a “hot zone” for conscious functions. However, neuroimaging and lesion studies suggest that higher-order conscious functions such as metacognition may also engage a frontoparietal network (Rouault *et al.* 2018; Vaccaro and Fleming 2018).

A more complete understanding of the neural mechanisms of metacognition also requires insight into the underlying neurotransmitter systems. To date, very little is known about the pharmacology of metacognition. Recently, Hauser *et al.* (2017) revealed that blockade of noradrenaline led to increased metacognitive sensitivity with unchanged perceptual decision-making performance, whereas both perceptual discrimination and metacognition remained unaffected by dopamine blockade.

One neurotransmitter likely to mediate aspects of consciousness is the glutamatergic system. Antagonists at the *N*-methyl-D-aspartate (NMDA) glutamate receptor, such as phencyclidine or ketamine, provoke psychedelic states which are clearly distinct from a normal waking state of consciousness (Anis *et al.* 1983; Umbricht *et al.* 2002; Morris and Wallach 2014), characterized by dissociative experiencing including vigilance reduction, ego transcendence, disembodiment, and visual and sensory disturbances (Vlisides *et al.* 2018). The noncompetitive NMDA-receptor antagonist ketamine is dose-dependently used for the treatment of depression (Murrough *et al.* 2013) and general anesthesia (Kurdi *et al.* 2014; Sarasso *et al.* 2015); in addition, it is a well-established research tool with an excellent safety record in both clinical and experimental applications (Javitt *et al.* 2012; Doyle *et al.* 2013). Ketamine-induced psychotropic effects such as distorted sense of space and time, euphoria and out-of-body experiences have contributed to its abuse as a recreational drug (Schifano *et al.* 2008; Giorgetti *et al.* 2015). Based on findings that acute ketamine administration temporarily and reversibly induces a range of both positive (hallucinations, thought disorder, delusions) and negative (social withdrawal, emotional blunting) psychosis-like symptoms in otherwise healthy volunteers, the compound is also a widely used pharmacological model of schizophrenia (Krystal *et al.* 1994; Malhotra *et al.* 1996).

Ketamine effects on cognition include a selective degradation of episodic memory (Hetem *et al.* 2000; Morgan *et al.* 2004). In episodic memory tasks, participants typically encode word items, and later retrieve those items by writing down as many words as they can remember (*recall*) or indicate whether a given item had previously been encoded or not (*recognition*) (Honey *et al.* 2005b). Previous findings suggest that retrieval performance is disturbed when ketamine is administered during encoding but remains unimpaired when only recognition, but not encoding, takes place under the influence of ketamine (Oye *et al.* 1992; Hetem *et al.* 2000; Honey *et al.* 2005b). This effect may, however, also depend on the depth of semantic processing of the encoded items: Honey *et al.* (2005b) found that ketamine reduced retrieval performance only when items were encoded at an intermediate level of processing (LoP), not on deep or shallow levels. A functional magnetic resonance imaging (fMRI) study by Honey *et al.* (2005a) reported that ketamine affects brain function during retrieval even if encoding occurred prior to ketamine administration: ketamine was associated with attenuated left prefrontal cortical response to deeply encoded items, whereas anterior cingulate activation was reduced for incorrect compared to correct responses.

Even though growing research effort is directed towards identifying the neural underpinnings of metacognition, and previous studies have aimed at specifying the role of glutamate in various cognitive functions, the involvement of this neurotransmitter system in metacognition has not yet been examined. In this double-blind, placebo-controlled fMRI study, the primary aim was to investigate the role of the glutamate system in metacognition and its underlying neural activity by applying a psychotomimetic dose of ketamine. Confidence ratings were collected in an episodic memory framework, based on the dissociation of ketamine effects on encoding and retrieval as operationalized by Honey *et al.* (2005a).

Specifically, we applied a task in which differences in Type 2 responses should not be due to altered Type 1 performance, since ketamine was previously shown to leave episodic memory performance in deep and shallow encoding conditions unaffected (Honey *et al.* 2005b). Metacognitive sensitivity was quantified using the meta-d′-framework, which was previously shown to be sensitive to the effects of pharmacological challenges (Clos *et al.* 2019) and drug consumption (Sadeghi *et al.* 2017). We expected metacognitive sensitivity to be altered by ketamine in both study phases and further predicted ketamine to affect neural activity during both metacognitive confidence ratings and encoding. The secondary study aims included investigation of LoP effects on retrieval performance and metacognitive accuracy as well as confirmation of the subjective, phenomenological effects of ketamine by including a self-report measure of altered states of consciousness.

## Materials and Methods

### Participants

Fifty-three healthy, non-smoking, right-handed volunteers (aged 18–34, *M *=* *23.47, SD* *=* *3.24; 29 female) with normal or corrected to normal vision and native speaker level command of German language were recruited for this study. Exclusion criteria were as follows: prior experience with ketamine, history of psychiatric or neurological disorder, claustrophobia, metalliferous implants, pregnancy, positive drug test, under- or overweight (Body Mass Index: <17; ≥30), or consumption of any medication. Further medical contraindications for the administration of ketamine included hypertension and hyperthyroidism.

The study was approved by the Research Ethics Committee at the Department of Psychology, University of Bonn (approval number: 18-03-28). In accordance with this approval, data of the study are not stored on public repositories, but behavioral data are available as Supplementary materials, and fMRI data will be made available upon request. Materials, analysis scripts, and preregistration of the study are available in Open Science Framework (https://osf.io/numxs/).

### Screening procedure

An online prescreening interview was conducted with individuals who responded to study advertisements. Those who met all inclusion criteria were invited for a screening visit in the laboratory, where the German version of the 5.0.0 MINI-International Neuropsychiatric Interview (Ackenheil *et al.* 1999), a urine drug screen (Drug-Screen Multi-5T, nal von minden GmbH) and, for females, a pregnancy test (NADAL hCG Pregnancy Test, nal von minden GmbH) were carried out to screen for exclusion criteria of psychiatric illness, drug abuse, and pregnancy. Measurements of height, bodyweight, and blood pressure were obtained. A medical questionnaire was used to exclude any current or past medical conditions, or any diagnosis of psychotic disorders among first-degree relatives. Additionally, the first five questions of the Structured Instrument for Prodromal Syndromes (SIPS 5.0) were included to rule out prodromal symptoms of schizophrenia (McGlashan *et al.* 2001). Suitable individuals were invited for assessment visits.

### Study design

A double-blind, randomized, placebo-controlled between-subjects design was employed. Randomization lists were created independently for females and males. The study team carrying out the assessments was not involved in the process of randomization. An unblinded study anesthesiologist prepared the infusion solution and constantly monitored oxygen saturation and heart rate of the participants during the infusion. Twenty-four participants were administered a subanesthetic dose of racemic ketamine (Ketamin-Ratiopharm 500 injection solution, Ratiopharm, Ulm, Germany), 29 participants received a saline solution (0.9% sodium chloride).

Ketamine was administered as a 2 mg/ml solution with a constant target plasma level of 100 ng/ml by a bolus and continuous infusion using a computerized infusion pump (Graseby 3500, Smith Medical Int. Ltd, Luton, UK). The solutions were administered using the STANPUMP program (Steven Shafer, M.D., Anesthesiology Service, PAVAMC 3801 Miranda Ave., Palo Alto, USA) based on the three-compartment model described by Domino *et al.* (1982). Previous studies of our group (Steffens *et al.* 2016, 2018) using the same infusion equipment and procedure confirmed that ketamine concentrations were close to the targeted plasma level and no residual traces of ketamine solution from the infusion site contaminated the results; therefore, no blood samples were drawn in this study.

### General procedure

On assessment days, participants were required to refrain from solid food for 6 h and clear fluids for 2 h before the infusion. Within 24 h before, participants were also instructed to take no medication and to stay abstinent from alcohol. Female participants took another pregnancy test on the day of assessment. After participants arrived, they completed the first study task (see below) before an additional medical screening was performed by the study anesthesiologist. Participants were then fitted with intravenous access into the nondominant arm and positioned in the MRI scanner. Following an individual adjustment of the field of view and an initial high-resolution structural imaging scan, the infusion was started.

Ketamine effects on metacognition, encoding, and retrieval in an episodic memory task were assessed in two separate study-test phases. Stimuli were selected from the Berlin Affective Word List (Võ *et al.* 2009); word class, frequency, emotionality, arousal level, number of syllables, and vividness were counterbalanced between conditions.

**Figure 1.**
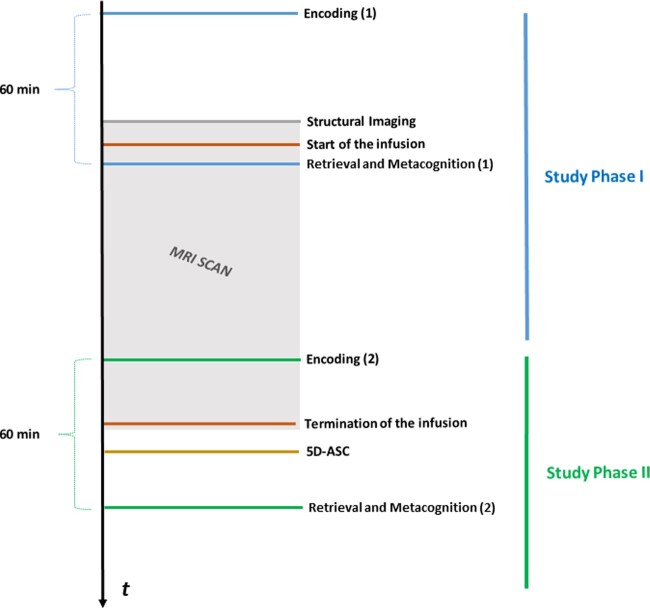
Study protocol. In Study Phase I (shown in blue), participants first encoded word items in the absence of infusion and before entering the MRI scanner. After a medical screening (in purple), participants completed a structural scan (in gray). Following the start of the infusion (in brown), retrieval of encoded items and corresponding metacognitive confidence was tested. As participants were still undergoing infusion in the MRI scanner, in Study Phase II (in green), participants encoded a second word list which was later retrieved outside the scanner, after termination of the infusion. Questionnaire data were collected using the 5D-ASC (in yellow). The MRI scanning period is represented by the grey box.

In Study Phase I, items were presented on a computer screen outside the MRI scanner, prior to drug infusion. Retrieval was tested ∼60 min after the end of the first encoding task, while BOLD data were acquired during infusion. In this first retrieval task, participants responded to stimuli by categorizing them either as “old items”, if they had previously been presented in the encoding task, or “new items”, if they had not been presented, and afterwards reported their metacognitive confidence (Type 2 response). Subsequently, in Study Phase II, another word list consisting entirely of novel items was encoded, as participants were still undergoing infusion in the MRI scanner. Retrieval of these items was tested ∼60 min after the infusion was terminated and participants had left the scanner. Immediately upon leaving the scanner, participants completed the 5D-ASC questionnaire to assess altered states of consciousness (Dittrich 1998). In the second retrieval task, items encoded in the second encoding task (“old items”) were again presented on a computer screen alongside “new items”, again requiring participants to state their confidence after each Type 1 response. Figure 1 provides an overview of the general procedure of assessment days.

### Task design

#### Study Phase I

Participants were presented with a total of 120 word items displayed in the center of a computer screen and were instructed to make one of two types of judgments about these items, which served as a manipulation of the depth of processing. We aimed for two levels of processing (deep/shallow) and selected a manipulation that could be expected to yield a pronounced LoP effect (Honey *et al.* 2005b). For each of 60 word items, participants indicated their subjective judgment of the pleasantness (pleasant/unpleasant) of the word (leading to deep encoding), whereas the other 60 items were encoded in a shallow manner, by participants reporting the number of syllables of each word (even/odd). Participants were not told that the retrieval of these items would be tested afterwards. These encoding tasks alternated blockwise, with each of four blocks comprising 30 items; the starting condition was determined randomly. Items were presented until keypress for a maximum of 3 s, with an interstimulus interval (ISI) of 0.5 s.

The fMRI retrieval task was implemented in an event-related design. Participants responded to items presented on the center of a monitor behind the MRI scanner via a mirror by predefined button presses. A total of 180 word items were used, including the 120 items that had been encoded in the previous task as well as 60 new items. The 2:1 ratio of old to new items was based on previous studies (Honey *et al.* 2005a). Items were presented in randomized order for a duration of 2.5 s followed by an ISI that varied randomly between 2 s and 6 s; participants were instructed to respond to items which they considered to be old, i.e., having previously been presented, with a left index finger button press and to items which they labeled as new with a right index finger button press.

**Figure 2.**
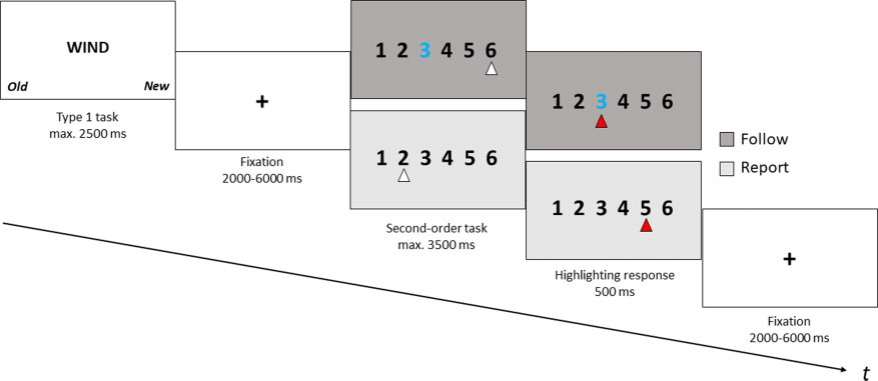
Schematic trial representation for the first retrieval task (stimuli are not to scale). Each trial consisted of two parts: first, participants categorized a presented word stimulus either as old (presented in the previous encoding task) or new (not having been presented before) (Type 1 task). Subsequently, they either indicated their subjective confidence (“Report” condition, shown in white) or placed the cursor at a color-coded position on the scale (“Follow” condition, grey) (Second-order task). The second retrieval task was similar, only here, the second-order task consisted entirely of “Report” trials, and the fixation period between task screens was shorter (1000 ms).

There were two types of second-order ratings: subsequent to 120 of these Type 1 responses, participants rated their subjective confidence regarding the judgment on a 6-point Likert scale (1 = “not confident at all”, 6 = “very confident”). In this “Report” condition, designed to tap metacognitive processes, participants moved a cursor along the scale, using their index fingers, until they reached the position on the scale that most accurately matched their subjective confidence, which they were instructed to confirm by a left or right thumb press. During the 60 “Follow” trials which served as a control condition not involving the actual process of confidence formation (Yokoyama *et al.* 2010; Fleming *et al.* 2012), participants were instructed to navigate the cursor towards a predefined number on the scale, highlighted in blue. The initial position of the cursor was random in each condition; there were no written labels to either point of the scale to avoid extreme responding bias (Overgaard *et al.* 2006). “Report” and “Follow” trials alternated in randomized order; exactly two-third of each of the episodic memory condition trials (deep/shallow/new) were followed by the “Report” condition. The duration of the decision window for this second-order response was 3.5 s, followed by a 0.5 s screen where a change in cursor color from white to red highlighted the participant’s response. Another variable ISI (2–6 s) preceded the onset of the next trial. In order to minimize exhaustion, the experiment was paused halfway through the task and a separate scan was started for the second half of the experiment. Figure 2 provides an illustration of the task.

#### Study Phase II

Following the completion of this first retrieval task, participants remained in the scanner and performed a second encoding task. Here, they were presented 100 novel word items in a block design; again, 50% of the items were encoded deeply by rating the subjective pleasantness of each word, whereas 50% of the items were encoded in a shallow manner by reporting the number of syllables. Again, encoding tasks alternated blockwise, with 10 blocks each comprising 10 items. At the beginning of each block, an instruction about the upcoming task was shown for 2 s. Participants responded via left or right button presses within a 3 s window (ISI = 0.5 s) for each item.

After termination of infusion and leaving the scanner, participants filled in the 5D-ASC, marking their extent of agreement with statements regarding various phenomenal experiences (Dittrich 1998). The 5D-ASC is a self-report questionnaire to retrospectively assess five dimensions of altered states of consciousness. These include three primary, etiology-independent scales, “Oceanic Boundlessness”, “Dread of Ego Dissolution”, and “Visionary Restructuralization”, which can be conflated to a global measure of altered consciousness, and two secondary, etiology-specific scales comprising further aspects of altered experiences, “Auditory Alterations” and “Vigilance Reduction”. 5D-ASC scale scores were formed following guidelines by Dittrich *et al.* (2006).

One hour after completion of the second encoding task, retrieval of those items was tested in a second retrieval task, without infusion at a time when plasma levels of ketamine are significantly reduced (Honey *et al.* 2005b). The design of the second retrieval task was almost identical to the first one, with two exceptions: ISI was constant (1 s), and there was no “Follow” condition, so participants had to report their confidence on each of the 150 trials (100 old, 50 new).

### fMRI data acquisition and analysis

Imaging was conducted using a 1.5 T Avanto MRI scanner (Siemens, Erlangen, Germany). High-resolution structural images were acquired to optimize normalization of functional imaging data using a T1-weighted 3D MPRAGE sequence [Repetition time (TR) = 1660 ms, echo time (TE) = 3.09, inversion time = 800 ms, matrix size = 256 × 256, slice thickness = 1.0 mm, FoV = 256 mm, flip angle = 15°, voxel size = 1 × 1 × 1 mm^2^, 160 sagittal slices]. Task-related BOLD fMRI data were acquired using a T2*-weighted echo-planar imaging sequence (TR = 2500 ms; TE = 45 ms, matrix size = 64 × 64, slice thickness = 3.0 mm, FoV = 192 mm, flip angle = 90°, voxel size = 3 × 3 × 3 mm, 31 slices). A standard 12-channel head coil was used for radio frequency transmission and reception.

fMRI data were analyzed using Statistical Parametric Mapping 12 software (Wellcome Centre for Neuroimaging, London, UK; http://www.fil.ion.ucl.ac.uk/spm) implemented in Matlab R2014a (The MathWorks Inc., Natick, USA). To allow for T1 equilibration, the first five volumes of each functional time series were discarded. Each participant’s structural image was segmented into gray matter, white matter, and cerebro-spinal fluid using a forward deformation field to map it onto template tissue probability maps (Ashburner and Friston 2005). Functional images were realigned to the first image of each time series to correct for head movement, using a six-parameter rigid body transformation. The realigned functional images were then coregistered to the anatomical image. For spatial normalization, functional scans were transformed into standard stereotaxic space of the Montreal Neurological Institute (MNI) template (Evans *et al.* 1992; Holmes *et al.* 1998) and resampled at 2 × 2 × 2 mm voxel size. Finally, images were spatially smoothed using an 8 mm full-width-at-half-maximum Gaussian kernel.

Following pre-processing, at the first (single-subject) level for Study Phase I, the onset of each stimulus was defined as the onset of the event; for Type 1 responses, the duration was set to be the reaction time from stimulus presentation to button press. For second-order responses, the function spanned the time from onset of scale presentation to the first movement participants made on the scale. This was done as the decisive metacognitive processes during Report trials were expected to take place during that time, and to eliminate motion-related activation. The realignment parameters were added to the model as covariates of no interest. Correctly retrieved deep, shallow and new items were included as Type 1 regressors; since there were too few cases of incorrect answers in the majority of participants, an overall residual regressor of no interest was formed for incorrect answers, thereby departing from our preregistered analysis plan.

Overall, there were four Type 1 regressors: “Deep” (*mean number of trials across participants*: 49, SD* *=* *8.6); “Shallow” (*M *=* *27.19, SD* *=* *11.34); “New” (*M *=* *47.02, SD* *=* *11.65); and “Incorrect” (*M *=* *41.85, SD* *=* *9.33). For each of these four regressors, two separate regressors were included for second-order ratings, resulting in a total of eight second-order regressors: “DeepReport” (*M *=* *29.92, SD* *=* *8.05); “DeepFollow” (*M *=* *17.68, SD* *=* *7.58); “ShallowReport” (*M *=* *18.96, SD* *=* *8.95); “ShallowFollow” (*M *=* *7.53, SD* *=* *4.7); “NewReport” (*M *=* *26.72, SD* *=* *6.69); “NewFollow” (*M *=* *13.17, SD* *=* *3.53); “IncorrectReport” (*M *=* *31.02, SD* *=* *9.04); “IncorrectFollow” (*M *=* *13.94, SD* *=* *5.03). All contrasts were estimated by comparing specific effects against the baseline of the respective first-level-model; consequently, the two separate runs were conflated in this step. Additionally, we set up an exploratory first-level-model, in which “Report” regressors were parametrically modulated by the selected confidence rating in each trial, all other regressors remaining unmodified, as only “Report” ratings were expected to require the engagement of metacognitive Type 2 evaluations.

For Study Phase II, the function spanned the time from onset of word presentation to button press. Here, a simpler model with conditions “Deep” and “Shallow” was specified. Also departing from preregistration, the factor “Retrieval Performance” (later correctly/incorrectly retrieved) could not be applied, as there was an insufficient amount of incorrect answers.

On the second level, a full factorial analysis was carried out on Study Phase I data using between-subjects factor “Drug” (ketamine/placebo) and within-subjects factor “Word Type” (deep/shallow/new) for Type 1 contrasts with an additional within-subjects-factor “Rating Type” (report/follow) for second-order contrasts. A separate full factorial analysis was conducted on Study Phase II data, using between-subjects-factor “Drug” (ketamine/placebo) and within-subjects-factor “Encoding Level” (deep/shallow).

All second-level analyses were conducted at the whole-brain-level. The statistical height threshold was *P* < 0.001, and significant clusters were inferred if the peak voxel of the cluster survived a statistical threshold of *P* < 0.05 family-wise-error (FWE) corrected (cluster-level). In order to assign anatomical labels, the anatomy toolbox was utilized (Eickhoff *et al.* 2005). To determine whether significant clusters of each contrast represented activations or deactivations, mean summary functions were created using MarsBaR (https://sourceforge.net/projects/marsbar).

BOLD data of four participants during Study Phase I and of three participants during Study Phase II had to be excluded from fMRI analysis because normalization failed. Consequently, fMRI data analysis was performed on 49 participants (23 ketamine, 26 placebo) for Study Phase I and on 50 participants (23 ketamine, 27 placebo) for Study Phase II. Behavioral data analysis was carried out on all 53 participants who completed the assessment.

### Behavioral data analysis

Following our preregistration, Type 1 (retrieval) and Type 2 (metacognitive) performance was assessed in an SDT framework (Green and Swets 1966; Barrett *et al.* 2013). We applied meta-d′ analysis (Maniscalco and Lau 2012) to quantify metacognitive *sensitivity*—i.e., the individual ability to discriminate between correct and incorrect retrieval judgments. Meta-d′ represents a response-bias free measure of how well confidence ratings track task accuracy and is on the same scale as the Type 1 sensitivity measure d′ (Maniscalco and Lau 2014). Meta-d′ was estimated in a maximum-likelihood-estimation model using code by Maniscalco (http://www.columbia.edu/~bsm2105/type2sdt) in Matlab R2016a (The MathWorks Inc., Natick, USA); only “Report” trials in which participants provided button presses on both retrieval and confidence rating were used for calculation. Additionally, metacognitive *efficiency* was calculated by dividing meta-d′ by d′ to provide an index of Type 2 performance that takes into account differences in Type 1 performance (Fleming and Lau 2014). To evaluate Type 2 performance, we therefore considered both absolute Type 2 sensitivity (meta-d′) and Type 2 efficiency relative to Type 1 performance (meta-d′/d′).

In addition to our preregistered analyses, we also conducted various exploratory analyses to facilitate mechanistic understanding of the outcomes. For example, we decided to expand our analysis to investigate ketamine effects on performance-corrected metacognitive bias (quantified as *mean judgment minus mean performance*) to test for differences in the selected confidence ratings between the two groups while controlling for the confounding influence of performance on confidence levels (Fleming and Lau 2014). Moreover, we explored Pearson’s correlations between Type 1 and both Type 2 performance measures as well as metacognitive bias in both study phases with the 5D-ASC global measure of altered consciousness; alpha-level was Bonferroni-corrected (*α*=.05/8=.006). Finally, we applied an extension of the HMeta-d toolbox (Fleming 2017), a hierarchical Bayesian estimation of metacognitive efficiency (https://github.com/metacoglab/HMeta-d) in Matlab R2016a, which estimates group-level parameters over log(meta-d′/d′) while taking into account uncertainty in model fits at the single-subject level. To test for a true group difference in metacognitive efficiency, we fitted separate models for the ketamine and placebo group and calculated the 95% highest-density intervals (HDIs; the interval containing 95% of the Markov chain Monte Carlo posterior samples) on the difference between the group posterior densities and evaluated their potential overlaps with zero (Kruschke 2014). We ran three chains for estimation and ensured chain convergence (Fleming 2017).

All other behavioral data analyses were conducted using SPSS 22 (IBM Corp., Armonk, USA). Data were tested for violation of statistical assumptions; Kolmogorov–Smirnov tests were applied to test for normality of distribution, Mauchly’s tests checked for sphericity, Levene’s statistics tested for homogeneity of variances and Box-M-tests for homogeneity of covariances. When normality was violated in only one variable of a group, none of the variables were transformed. Drug effects on 5D-ASC scales, Type 1 and Type 2 reaction times and metacognitive bias were tested via independent samples *t*-tests. Paired *t*-tests were employed to compare Type 1 and Type 2 reaction times and metacognitive bias between deeply vs. shallowly encoded items. Separate mixed-design ANOVAs were employed with factors “Encoding Level” and the “Drug” for Type 1 and Type 2 sensitivity and Type 2 efficiency. Effect sizes for *t*-tests are given in Cohen’s *d* (Cohen and Maydeu-Olivares 1992), effect sizes for ANOVAs in partial eta-squared (Cohen 1973).

## Results

### 5D-ASC

There was a significant ketamine effect on the 5D-ASC global measure of altered consciousness [*t*(23.7) = 4.69, *P* < 0.001, *d *=* *1.35] and on all scales. Participants who had received ketamine scored significantly higher on the three primary dimensions “Oceanic Boundlessness” [*t*(23.23) = 4.04, *P* < 0.001, *d *=* *1.17], “Dread of Ego Dissolution” [*t*(25.73) = 4.56, *P* < 0.001, *d *=* *1.31], and “Visionary Restructuralization” [*t*(23.43) = 3.48, *P* = 0.002, *d *=* *1.01]. They also achieved significantly higher values on the “Auditory Alterations” [*t*(28.17) = 4.55, *P* < 0.001, *d *=* *1.29] and “Vigilance Reduction” scales [*t*(34.01) = 5.99, *P* < 0.001, *d *=* *1.69]. Descriptive statistics are provided in Table 1.

**Table 1.** Descriptive statistics of 5D-ASC questionnaire scores by drug.

Scale	Placebo (*n* = 29)		Ketamine (*n* = 24)	
	*M*	SD	*M*	SD
[Global Index of Altered States]	1.08	1.87	14.51	13.91
Oceanic Boundlessness	0.71	1.48	16.63	19.25
Dread of Ego Dissolution	2.05	3.26	13.71	12.18
Visionary Restructuralization	0.52	1.74	12.27	16.45
Auditory Alterations	1.85	4.63	14.14	12.56
Vigilance Reduction	12.58	14.04	47.71	25.75

Note: Scale values are given in percent. M, mean; SD, standard deviation.

#### Exploratory analyses

There were no significant correlations of the 5D-ASC global measure of altered consciousness with Type 1 and Type 2 outcomes in either study phase (all *P* > 0.006).

### Study Phase I

Descriptive statistics of Type 1 and Type 2 measures for Study Phase I are provided in Table 2. Distribution plots of raw data for all relevant dependent variables can be found in the Supplementary materials.

**Table 2.** Descriptive statistics of study phase I sensitivity measures (type 1 and type 2) and reaction times (type 1 and type 2) by drug and encoding level.

Measure	Placebo (*n* = 29)		Ketamine (*n* = 24)	
	*M*	SD	*M*	SD
Type 1 performance (d')^a^				
Deep vs. new	2.11	0.63	1.94	0.49
Shallow vs. new	0.85	0.38	0.74	0.37
Type 2 sensitivity (meta-d')^a^^,b^				
Deep vs. new	2.41	0.95	2.06	0.72
Shallow vs. new	0.89	0.49	0.58	0.39
Type 2 efficiency (meta-d'/d')				
Deep vs. new	1.17	0.38	1.13	0.48
Shallow vs. new	1.15	0.61	0.92	0.69
Type 1 reaction times (in ms)^a^				
Deep	1415.66	216.69	1458.75	189.0
Shallow	1572.63	197.31	1576.0	174.66
New	1600.11	227.04	1549.02	152.33
Type 2 reaction times (in ms)^a^				
Deep	1592.22	210.58	1578.45	221.79
Shallow	1670.7	229.7	1698.43	297.13
New	1755.37	251.38	1731.03	284.39

M, mean; ms, milliseconds; SD, standard deviation.

^a^ Significant effects of encoding level.

^b^ Significant effects of drug.

#### Type 1 behavioral analyses

The LoP manipulation was successful: participants showed significantly enhanced retrieval performance for deeply compared to shallowly encoded items [main effect of “Encoding Level”: *F*(1,51) = 241.44, *P* < 0.001, *η*_p_^2^ = 0.83]. However, there was no main effect of “Drug” [*F*(1,51) = 1.78, *P* = 0.188, *η*_p_^2^ = 0.03]; ketamine did not significantly alter retrieval performance. Type 1 reaction times were significantly shorter for deeply than shallowly encoded items [*t*(52) = 9.17, *P* < 0.001, *d* = 0.71] but were unaffected by ketamine [*t*(51) = 0.04, *P* = 0.972, *d* < 0.01]. There were no significant interactions (*P *>* *0.05).

#### Type 1 fMRI analyses

For BOLD data during retrieval, there was no significant difference between ketamine and placebo (*P *>* *0.05). For a detailed summary of LoP and Old vs. New effects, see Supplementary materials.

#### Type 2 behavioral analyses

Participants showed enhanced metacognitive sensitivity for deeply compared to shallowly encoded items [*F*(1,51) = 186.36, *P* < 0.001, *η*_p_^2^ = 0.79]. Importantly, there was a significant main effect of “Drug” [*F*(1,51) = 4.64, *P* = 0.036, *η*_p_^2^ = 0.08]: metacognitive sensitivity deteriorated under ketamine. However, there was no significant main effect of either “Drug” [*F*(1,50) = 1.03, *P* = 0.315, *η*_p_^2^ = 0.02) or “Encoding Level” [*F*(1,50) = 2.17, *P* = 0.147, *η*_p_^2^ = 0.04] on metacognitive efficiency. Type 2 reaction times were faster for deeply encoded items [*t*(52) = 4.25, *P* < 0.001, *d* = 0.41] but were found to be unaltered by “Drug” [*t*(51) = 0.03, *P* = 0.98, *d* < 0.01]. There were no significant interactions (*P *>* *0.05).

##### Exploratory analyses

**Figure 3.**
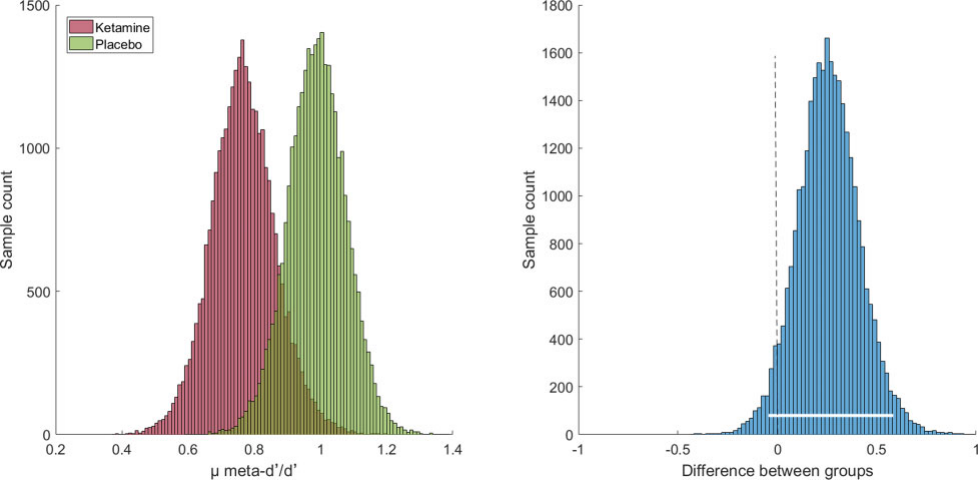
Hierarchical Bayesian estimation of metacognitive efficiency (µ_meta-d′/d′_) in Study Phase I. Left panel: Group-level values for the ketamine group (red histogram) and the placebo group (green histogram). Right panel: Difference in group posteriors (in log units). The white bar indicates the 95% highest-density interval which narrowly overlaps with zero.

Hierarchical Bayesian estimation of group-level meta-d′/d′ confirmed that we cannot be certain that there is a true difference in metacognitive efficiency between the two groups, even though the estimated difference between groups was relatively high [mean: 0.23 (highest-density interval: −0.04 to 0.58)]. Figure 3 provides an illustration of the estimated group-level parameters of metacognitive efficiency.

There was also a significant effect of “Drug” on metacognitive bias scores [*t*(51) = 2.15, *P* = 0.037, *d* = 0.59), with participants under ketamine being overconfident. In addition, there was a significant effect of “Encoding Level” on metacognitive bias, with ratings for shallowly encoded items reflecting overconfidence [*t*(48) = 7.25, *P* < 0.001, *d *=* *1.24].

#### Second-order fMRI analyses

##### Report vs. follow effects

**Figure 4.**
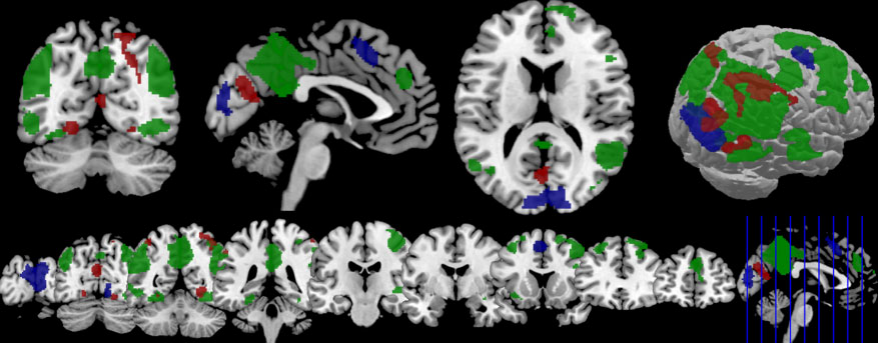
Second-order fMRI results. Significant activation in the contrasts Ketamine>Placebo (red), Report>Follow (blue) and Follow>Report (green) at significance level *P *<* *0.001 (uncorrected).

Higher BOLD responses during Report than Follow were found in a right visual cluster of right calcarine and lingual gyrus (Figure 4, Table 3). The cluster furthermore encompassed left and right cuneus, as well as bilateral superior occipital gyrus. A second, left-hemispheric, cluster was located in the posterior medial frontal cortex (pMFC).

**Table 3.** Summary of significant clusters for the report > follow contrast.

Anatomical label	Laterality	Cluster size [k]	*T*-value	Peak voxel MNI coordinates		
Calcarine gyrus	R	1599	5.33	8	−86	4
Lingual gyrus	R		5.24	12	−80	−8
Cuneus	R		4.88	8	−86	26
Cuneus	L		4.61	−6	−94	22
Superior occipital gyrus	L		4.58	−10	−96	20
Superior occipital gyrus	R		3.81	18	−96	18
pMFC	L	352	6.08	−4	16	48

Combined sample. Only unique anatomical labels are reported for each cluster at one laterality. Whole-brain cluster-level FWE corrected (*P *<* *0.001 uncorrected).

FWE, family-wise error; L, left; MNI, Montreal Neurological Institute; pMFC, posterior medial frontal cortex; R, right.

The reverse effect (Follow>Report, indicating BOLD responses that were higher when participants had to select a predefined specification on the scale) revealed a total of 11 clusters (Figure 4, Table 4). These correspond to the default-mode network (DMN) that is active in the absence of task demands (Andrews-Hanna 2012), which encompasses angular gyrus, precuneus, posterior cingulate cortex (PCC), superior frontal areas, and parahippocampal gyrus, all of which were activated in the contrast.

**Table 4.** Summary of significant clusters for the follow > report contrast.

Anatomical label	Laterality	Cluster size [k]	*T*-value	Peak voxel MNI coordinates		
Angular gyrus	R	3662	9.36	56	−52	36
Superior parietal lobule	R		4.8	36	−44	58
Middle occipital gyrus	R		4.53	36	−80	10
Postcentral gyrus	R		3.36	24	−44	66
Precuneus	R	3384	7.18	10	−50	38
PCC	R		6.96	4	−48	28
MCC	R		6.85	10	−44	32
Precuneus	L		5.46	−6	−52	44
MCC	L		5.11	−4	−46	48
Superior frontal gyrus	R	2630	7.22	4	46	30
Middle frontal gyrus	R		5.54	30	24	54
IFG (p. Triangularis)	R		4.53	48	24	24
Inferior parietal lobule	L	2548	7.14	−54	−54	36
Angular gyrus	L		5.97	−40	−72	38
Supramarginal gyrus	L		4.37	−62	−36	38
Middle occipital gyrus	L		4.05	−36	−80	28
Fusiform gyrus	L	1094	7.34	−30	−52	−16
Inferior temporal gyrus	L		4.61	−54	−54	−8
Middle temporal gyrus	L		4.07	−60	−50	−2
Parahippocampal gyrus	L		3.32	−22	−28	−18
Fusiform gyrus	R	807	7.5	30	−52	−16
Inferior occipital gyrus	R		5.35	36	−72	−10
Inferior temporal gyrus	R		3.88	52	−64	−8
Precentral gyrus	R	753	5.67	38	−22	54
Middle temporal gyrus	R	682	5.5	60	−20	−10
Posterior insula	R		4.49	34	−6	−12
Insula lobe	R		4.41	40	−18	−2
Superior temporal gyrus	R		3.45	50	−12	−10
Middle frontal gyrus	L	674	5.57	−32	24	50
Superior frontal gyrus	L		4.3	−22	22	56
Superior frontal gyrus	R	203	4.48	14	66	16
Anterior insula	L	162	4.99	−28	6	−14
Insula lobe	L		4.49	−32	16	−12

Combined sample. Only unique anatomical labels are reported for each cluster at one laterality. Whole-brain cluster-level FWE corrected (*P *<* *0.001 uncorrected).

FWE, family-wise error; IFG, inferior frontal gyrus; L, left; MCC, midcingulate cortex; MNI, Montreal Neurological Institute; PCC, posterior cingulate cortex; R, right.

##### Drug effects

During second-order ratings (both Report and Follow), there was larger BOLD with ketamine than placebo in five clusters (Figure 4, Table 5): The first, right-hemispheric, cluster included superior parietal lobule (SPL), supramarginal gyrus, inferior parietal lobule (IPL), and angular gyrus. A second cluster was located in left calcarine gyrus, a third cluster in right lingual gyrus. The fourth cluster included left IPL, whereas a fifth, left-hemispheric cluster encompassed lingual gyrus and fusiform gyrus. There were no significant effects for the reverse contrast and no significant interactions (*P *>* *0.05).

**Table 5.** Summary of significant clusters for the ketamine > placebo contrast.

Anatomical label	Laterality	Cluster size [k]	*T*-value	Peak voxel MNI coordinates		
Superior parietal lobule	R	642	5.51	36	−52	64
Supramarginal gyrus	R		3.56	60	−28	48
Middle occipital gyrus	R		3.32	30	−64	33
Inferior parietal lobule	R		3.26	40	−54	48
Angular gyrus	R		3.23	36	−56	48
Calcarine gyrus	L	257	4.59	−2	−72	18
Lingual gyrus	R	212	4.42	18	−70	−10
Inferior parietal lobule	L	188	4.24	−40	−52	60
Lingual gyrus	L	172	5.21	−18	−68	−8
Fusiform gyrus	L		3.98	−28	−52	−12

Only unique anatomical labels are reported for each cluster at one laterality. Whole-brain cluster-level FWE corrected (*P *<* *0.001 uncorrected).

FWE, family-wise error; L, left; MNI, Montreal Neurological Institute; R, right.

##### Exploratory analyses

Parametric modulation analysis (“Report” trials parametrically modulated by the selected confidence rating) revealed very similar results, i.e., higher BOLD response for ketamine than placebo in bilateral lingual, fusiform, and calcarine gyrus and right SPL (see Supplementary Table 6). There were no significant effects for the reverse contrast and no significant interactions (*P *>* *0.05).

### Study Phase II

#### Encoding: fMRI analyses

There were no significant ketamine effects on BOLD during encoding (*P *>* *0.05). For LoP effects, see Supplementary materials.

#### Type 1 behavioral analyses

Descriptive statistics of Type 1 and Type 2 measures for Study Phase II are provided in Table 6. Distribution plots of raw data for all relevant dependent variables can be found in the Supplementary materials.

**Table 6.** Descriptive statistics of study phase II sensitivity measures (type 1 and type 2) and reaction times (type 1 and type 2) by drug and encoding level.

Measure	Placebo (*n* = 29)		Ketamine (*n* = 24)	
	*M*	SD	*M*	SD
Type 1 performance (d')^a^				
Deep vs. new	1.79	0.58	1.59	0.54
Shallow vs. new	0.68	0.41	0.57	0.31
Type 2 sensitivity (meta-d')^a^				
Deep vs. new	1.97	0.78	1.77	0.68
Shallow vs. new	0.44	0.41	0.4	0.58
Type 2 efficiency (meta-d'/d')^a^				
Deep vs. new	1.08	0.35	1.2	0.6
Shallow vs. new	0.69	0.65	0.71	1.19
Type 1 reaction times (in ms)^a^				
Deep	1267.99	235.79	1233.3	187.04
Shallow	1368.44	245.62	1306.19	186.04
New	1416.41	268.68	1286.1	187.95
Type 2 reaction times (in ms)^a^				
Deep	1132.52	213.1	1077.98	300.41
Shallow	1194.43	253.64	1112.9	301.18
New	1228.7	290.89	1117.47	272.8

M, mean; ms, milliseconds; SD, standard deviation.

^a^ Significant effects of encoding level.

Items that had been encoded deeply were recognized more often than shallowly encoded items [significant main effect of “Encoding Level”: *F*(1,51) = 273.94, *P* < 0.001, *η*_p_^2^ = 0.85]. There was no significant effect of “Drug” on d′ [*F*(1,51) = 1.8, *P* = 0.185, *η*_p_^2^ = 0.04]. “Drug” also had no effect on Type 1 reaction times [*t*(51) = 1.29, *P* = 0.203, *d* = 0.36]; when deeply encoded items were presented, participants made significantly quicker button presses [*t*(52) = 5.7, *P* < 0.001, *d* = 0.4]. There were no significant interactions (*P *>* *0.05).

#### Type 2 behavioral analyses

There were significant main effects of “Encoding Level” on metacognitive sensitivity [*F*(1,50) = 263.38, *P* < 0.001, *η*_p_^2^ = 0.84] and metacognitive efficiency [*F*(1,49) = 18.01, *P* < 0.001, *η*_p_^2^ = 0.27), but no effects of “Drug” on either meta-d′ [*F*(1,50) = 0.655, *P* = 0.422, *η*_p_^2^ = 0.01] or metacognitive efficiency [*F*(1,49) = 0.16, *P* = 0.691, *η*_p_^2^ < 0.01]. Type 2 reaction times were also significantly shorter for deeply encoded items [*t*(51) = 2.68, *P* = 0.01, *d* = 0.19], but there was no effect of “Drug” [*t*(50) = 1.13, *P* = 0.264, *d* = 0.34). There were no significant interactions (*P *>* *0.05).

##### Exploratory analyses

Corresponding to overlaps of 95% HDIs with zero, we found no significant group difference in metacognitive efficiency between ketamine and placebo [0.03 (−0.35 to 0.043)]. Thus, there was no significant ketamine effect on any measure of Type 2 performance when retrieval took place after termination of the infusion. We did, however, observe a significant effect of “Drug” on metacognitive bias [*t*(50) = 2.75, *P* = 0.008, *d* = 0.76], with participants under ketamine displaying overconfidence. There was also significantly larger metacognitive bias for shallowly than for deeply encoded words [*t*(50) = 9.31, *P* < .001, *d *=* *1.33].

## Discussion

This study investigated the role of the glutamate system in metacognition and associated brain activity using a ketamine challenge during episodic memory tasks in two study phases.

### Study Phase I

#### Task effects

For a detailed discussion on LoP effects both at the behavioral and the brain functional level, see Supplementary materials.

Two clusters were significantly more active during Report than Follow; the first includes right calcarine gyrus, bilateral cuneus, and right lingual gyrus. The latter structure has been demonstrated to display increased functional connectivity with prefrontal cortex (PFC) in Report compared to Follow trials (Fleming *et al.* 2012). The second cluster in left pMFC provides further evidence for its role in metacognition and resembles the anatomically adjacent dorsal anterior cingulate cortex cluster which Fleming *et al.* (2012) found to be involved in reporting confidence in a similar task design. A recent meta-analysis (Vaccaro and Fleming 2018) identified bilateral pMFC as one of the prime neural correlates of metacognitive judgments, representing the biggest cluster in the composite meta-analysis of all metacognition-related activity and the second-biggest cluster associated with metacognitive ratings following memory judgments.

In the reverse contrast (Follow>Report), increased activation was found in brain regions involved in the DMN, which has been linked to introspective mental activities such as mind wandering (Andrews-Hanna 2012). Again, this confirms Fleming *et al.* (2012), who reported similar patterns in this contrast.

#### Drug effects

As expected, subjective measures (5D-ASC) revealed that ketamine caused phenomenological experiences significantly deviating from a normal state of consciousness on all scales of the questionnaire. This finding confirms the known psychotomimetic effects of ketamine (Anis *et al.* 1983; Vlisides *et al.* 2018) and validates the rationale for using this pharmacological challenge to investigate the glutamatergic basis of metacognition.

Our study is one of only very few to indicate a potential pharmacological modulation of metacognitive performance (Lou *et al.* 2011; Hauser *et al.* 2017) and the first to investigate ketamine effects on metacognition. We show that disrupting the glutamatergic system by means of ketamine administration may challenge introspective monitoring processes: at the behavioral level, ketamine application during retrieval resulted in deterioration of metacognitive sensitivity (meta-d′) and overconfidence (larger metacognitive bias). Differences in metacognitive bias have been suggested to reflect genuine differences in awareness (Schwiedrzik *et al.* 2011), suggesting a role of various conscious processes giving rise to this ketamine effect on metacognitive bias. Furthermore, as overconfidence has been reported in patients with schizophrenia (Moritz *et al.* 2014), this finding provides another piece of evidence for use of ketamine as a model system of schizophrenia. Importantly, ketamine did not affect retrieval (Type 1) performance, in line with previous reports (Honey *et al.* 2005b), even though some group-heterogeneity has to be considered in Type 1 performance. Additionally, both Type 1 and Type 2 reaction times were unaffected by ketamine, which also indicates that the drug did not lead to a general deterioration of cognitive performance.

However, when controlling for the influence of Type 1 performance (d′) on metacognitive sensitivity (meta-d′) by calculating metacognitive efficiency (meta-d′/d′), there was no significant group difference. It is advised to apply metacognitive efficiency measures when comparing different groups (Fleming and Lau 2014; Vaccaro and Fleming 2018) although the theoretical assumption of the relationship of Type 1 and Type 2 performance measures (Galvin *et al.* 2003; Maniscalco and Lau 2012) is frequently violated in cases of “hyper”-metacognitive efficiency (meta-d′/d′ > 1), potentially arising as a consequence of post-decisional and/or second-order computation (Fleming and Daw 2017) as evidence continues to be accumulated after the Type 1 response (Murphy *et al.* 2015; Rausch and Zehetleitner 2016). In general, meta-d′ represents a measure of an individual’s ability to discriminate between their own correct and incorrect responses independently of differences in response bias (Fleming and Lau 2014) and prior studies have reported meta-d′ either as the only measure of metacognitive sensitivity (Rausch *et al.* 2015) or alongside the meta-d′/d′ ratio (Beck *et al.* 2019).

While it is necessary to keep in mind that the ketamine-associated deterioration of Type 2 sensitivity might be influenced by non-significant group-heterogeneity in Type 1 performance, rather than reflecting a general deficit in the underlying metacognitive processes (Maniscalco and Lau 2012), it is still important to understand ketamine effects on meta-d′ in Study Phase I. This is based on the absence of group effects on Type 1 performance in our study but also on the fact that 95% HDIs only narrowly overlapped with zero in two-sided testing for group differences in metacognitive efficiency. The group-level estimation in a hierarchical Bayesian framework offers several methodological advantages over previous estimation methods for metacognitive efficiency (Fleming 2017). As illustrated in Figure 3, there was an almost perfect fit of the ideal observer model in the placebo group (group-level meta-d′/d′: 0.99), whereas the ketamine group (0.76) substantially deviated from the ideal observer model implied in the meta-d′-framework (Fleming 2017).

Furthermore, we observed a pronounced up-regulation of activity in posterior brain regions with ketamine. This effect was observed only during second-order ratings (including both metacognitive reports and the control condition), whereas Type 1 BOLD showed no difference in activation between the groups. Specifically, there was increased activity in the right-hemispheric superior-posterior cortex compared to placebo. The superior parietal lobe is mainly associated with spatial attention and plays a pivotal role in somatosensory and visuomotor integration (Culham and Valyear 2006; Iacoboni 2006), motor learning (Weiss *et al.* 2003; Wenderoth *et al.* 2004), mental rotation (Wolbers *et al.* 2003; Gogos *et al.* 2010), with a mosaic of specialized subregions (Wang *et al.* 2015). Increased BOLD with ketamine also occurred in left calcarine gyrus, where the primary visual cortex is concentrated (Goebel *et al.* 1998; Seghier *et al.* 2000); bilateral lingual gyrus, which has been linked to processing vision (especially letter-reading) and encoding visual memories (Mechelli *et al.* 2000); and left IPL, which is involved in language processing, mathematical operations and body image (Radua *et al.* 2010), agency (Chaminade and Decety 2002), and working memory (Ravizza *et al.* 2004). Importantly, these ketamine effects on BOLD were observed for both second-order rating types (Report/Follow) and are therefore not specific to genuine metacognitive processes. It should be noted, however, that Report trials were overall more frequent (2:3) than Follow trials and thus had a greater overall contribution to the ketamine effects on second-order BOLD.

Overall, it appears that ketamine affects brain function during second-order ratings by means of an up-regulation of posterior visuospatial cortical brain areas. The visual, affective word stimuli employed in this study may have evoked vivid, imaginative processes in all participants, irrespective of drug, during retrieval. In participants experiencing the altered state of consciousness induced by ketamine, these imaginative processes may yet have persisted well beyond the retrieval process and consequently perturbed the signal available for the second-order task, irrespective of its specific demands, which could account for both the deterioration in metacognitive sensitivity as well as the increased activation in visuospatial areas during second-order ratings. However, it should be reiterated that it is uncertain to what extent the observed effects are related to metacognition, or whether they do not simply reflect neural responses to the presentation of the rating scale.

It is intriguing, however, that the anatomical location of our results is of interest with regards to the “hot zone” for conscious functions proposed by Koch *et al.* (2016): As this hot zone primarily encompasses sensory areas, it is mainly associated with phenomenal qualities of conscious experiences, which self-reported 5D-ASC measures confirmed to be altered by ketamine. Thus, as individuals under the influence of ketamine processed the demands of the second-order task (including introspective assessments of their internal mental world), phenomenal qualities of their normal waking-state experience may be distinctly altered. The posterior parietal cortical areas found in this study have been proposed to encode decision confidence (Kiani and Shadlen 2009), but recent studies suggest that activity in these areas tracks reliability of the sensory input rather than the core process of confidence formation (Bang and Fleming 2018). Accordingly, our findings suggest that not confidence formation itself, but early aspects of the metacognitive process could be impacted by ketamine as individuals struggle to make sense of a distorted input signal which results in an up-regulation of neural activity, whereas episodic memory or processing speed remain largely unaffected.

This interpretation is supported by evidence that ketamine increases bilateral temporoparietal functional connectivity (Höflich *et al.* 2015) and causes a significant alpha current reduction in posterior cortical areas such as precuneus and temporoparietal junction, which may reflect efforts to maintain ego integrity (Carhart-Harris *et al.* 2014; Vlisides *et al.* 2018). The ketamine-induced psychedelic state is characterized by elevated entropy in certain aspects of brain function, thereby collapsing the highly organized, low-entropy activity within the DMN (Carhart-Harris *et al.* 2014). This is in line with the notion by Carhart-Harris *et al.* (2014) that DMN integrity is a key foundation for accurate metacognition: upon perturbing DMN activity by inducing a psychedelic state, the functionality of metacognitive processes should hence be reduced, whereas the retrieval process may in many cases be based on a notion of familiarity with the word item, and therefore depend less on DMN integrity.

To achieve a comprehensive understanding of the findings, there are additional aspects to be considered. First, the lack of correlation between the 5D-ASC index of altered consciousness and ketamine effects on metacognitive sensitivity makes it difficult to draw a direct connection between the ketamine-induced altered subjective state and the observed objective effects on metacognition—although it may not be adequate to assume both effects to take place on the same conscious level, since the impairment of metacognition represents unconscious effects on conscious decisions (such as ratings given on the 5D-ASC). Second, it has to be considered that different causes might result in a deterioration of metacognitive sensitivity. Both a reduction in the sensory reliability of the input to the metacognitive process (i.e. increased noise in the evidence on which confidence formation is based) as well as trial-to-trial variability in the placement of confidence criteria might account for this effect. A clear interpretation remains difficult, but exploratory analysis of metacognitive bias, which revealed significantly higher bias (i.e. overconfidence) for the ketamine group, offers potential insights into the underlying mechanisms: fluctuations across individual trials in participants’ confidence indicate that participants under the influence of ketamine based their confidence ratings on certain conscious experiences, which could be due to changes in conscious access as well as altered, hallucinatory-like experiences, and which are ultimately unknown to the experimenter (Fleming and Lau 2014). Ultimately, it is possible that the unspecific up-regulation of the posterior parietal areas during second-order ratings reflect either the disturbances in signal input or alterations in conscious experience, or even both.

### Study Phase II

#### Drug effects

There were no ketamine effects on Type 1 sensitivity or Type 2 sensitivity and efficiency of items encoded during maintained drug infusion. This was confirmed by exploratory hierarchical Bayesian estimation of group-level metacognitive efficiency; unexpectedly, there was no group difference in metacognitive performance for Study Phase II. The absence of ketamine effects on retrieval is in accordance with previous studies (Honey *et al.* 2005a,b) using a very similar LoP manipulation. We found no drug-related group differences in functional activity during encoding in the continued presence of drug infusion and were thus unable to reproduce the increased activation for deeply encoded items in left PFC with ketamine reported by Honey *et al.* (2005a). Moreover, there were no effects of ketamine on either Type 1 or Type 2 reaction times, again indicating that ketamine did not affect reaction speed. However, metacognitive bias (overconfidence) was again significantly higher in the ketamine group, as was the case in Study Phase I. Even when ketamine was absent at retrieval, ketamine participants were overconfident about their mnestic judgments, suggesting that ketamine evokes substantial distortions in the placement of confidence criteria, irrespective of whether encoding or retrieval took place under the influence of ketamine. While it not possible to retroactively rule out a baseline difference in confidence level between the groups, an overall diffuse memory trace might account for the observed overconfidence, as ketamine affects source memory (Honey *et al.* 2005b). Therefore, ketamine effects on metacognitive bias could be driven by shared and distinct mechanisms for the two study phases.

### Limitations

The employment of a between-subjects-design might be a potential shortcoming, as homogeneity in all relevant individual factors can never be achieved across the groups. However, the advantage of this design is that expectancy biases based on experience with the first of two assessments in a within-subjects-design are eliminated.

Whilst the infusion protocol served to keep plasma levels of ketamine constant, it cannot be ruled out that participants became accustomed to the ketamine-induced state of consciousness and developed mechanisms to stabilize higher-order cognitive functions over the course of the infusion. This potential habituation effect may account for the observation that encoding processes in Study Phase II were less affected by ketamine than previously observed (Honey *et al.* 2005a,b).

As participants were not informed about the subsequent retrieval task at encoding in either study phase, it is important to point out that during the encoding task in Study Phase II, participants might have been more likely to infer the subsequent memory testing, which could have altered their encoding strategy. This introduces an additional difference between the two study phases, which complicates a direct comparison of ketamine effects between the phases.

Another limitation is that only trials with correctly retrieved items could be included in the fMRI analyses, due to the fact that the majority of participants produced an insufficient amount of incorrect answers in the Type 1 task. Finally, even though the combined sample size of both groups corresponded to sample sizes of previous within-subject designs (Steffens *et al.* 2016, 2018; Van Loon *et al.* 2016), it is possible that the study lacked sufficient power to detect a statistically significant difference between groups not only on metacognitive sensitivity but also on efficiency.

Generally, additional research is required to gain further understanding of ketamine effects on metacognition. Such potential future research efforts could encompass the application of advanced modeling capable of contrasting theories, such as the Stochastic Detection and Retrieval Model (Jang *et al.* 2012), which could help disentangle the underlying mechanisms of the observed effects and allow to discriminate between increased noise in the sensory evidence accumulation and trial-by-trial variability in the placement of confidence criteria. Furthermore, dynamic causal modeling of fMRI results could also help to clarify the extent to which the vivid, imaginative processes affect brain activity during second-order ratings.

## Conclusions

In summary, we present evidence for a role of the NMDA-glutamate-receptor antagonist ketamine in metacognition, including significantly larger metacognitive bias and deterioration of metacognitive sensitivity with ketamine. We also observed unspecific up-regulation of activity in posterior brain areas during second-order ratings compared to placebo. Importantly, ketamine did not affect metacognitive efficiency as estimated in a hierarchical Bayesian framework. The reported effects are neither sufficiently strong nor specific enough to attribute metacognition solely to the function of the glutamatergic system. Our results do, however, suggest that ketamine impacts on metacognition, which could be due to a reduction in the sensory reliability of the input to the metacognitive process as well as alterations in conscious experience. Further research is required in order to expand our understanding of the neural and pharmacological underpinnings of metacognition.

## Supplementary Material

niaa028_Supplementary_Data
