# Supplementary material for: Effects of ketamine on brain function during metacognition of episodic memory
Source: Neurosci Conscious. 2021 Feb 10;2021(1):niaa028. doi: 10.1093/nc/niaa028 (PMC7959215; doi:10.1093/nc/niaa028)
Supplement: niaa028_Supplementary_Data [file niaa028_supplementary_data.zip › Supplementary Materials.pdf]

Effects of Ketamine on Brain Function during Metacognition of Episodic Memory – Supplementary Materials

Mirko Lehmann (1), Claudia Neumann (2), Sven Wasserthal (3), Johannes Schultz (4, 5), Achilles Delis (2), Peter Trautner (4, 5, 6), René Hurlemann (3, 7, 8), Ulrich Ettinger (1)

(1) Department of Psychology, University of Bonn, Bonn, Germany

(2) Department of Anesthesiology, University Hospital Bonn, Bonn, Germany

(3) Department of Psychiatry and Division of Medical Psychology, University Hospital Bonn, Bonn, Germany

(4) Center for Economics and Neuroscience, University of Bonn, Bonn, Germany

(5) Institute for Experimental Epileptology and Cognition Research, University of Bonn Medical Center, Bonn, Germany

(6) Department for NeuroCognition, Life & Brain Center, Bonn, Germany

(7) Department of Psychiatry, School of Medicine & Health Sciences, University of Oldenburg, Oldenburg, Germany

(8) Research Center Neurosensory Science, University of Oldenburg, Oldenburg, Germany

Correspondence:

Ulrich Ettinger (ulrich.ettinger@uni-bonn.de); Department of Psychology, Kaiser-Karl-Ring 9, 53111 Bonn, Germany

Study Phase I: Results (Task Effects)

Type 1 fMRI Analyses

LoP effects: Deeply encoded items elicited larger BOLD than shallowly encoded items in six predominantly left-hemispheric clusters, including midcingulate cortex (MCC), precuneus, angular gyrus, middle temporal cortex, frontal cortex and hippocampus. There were no significant results for the reverse contrast (P>0.05).

Supplementary Table 1. Study Phase I, Type 1. Summary of significant clusters for the Deep > Shallow contrast.

Anatomical label	Laterality	Cluster size [k]	*T*-Value	Peak voxel MNI coordinates		
MCC	L	2465	9.36	-10	-46	36
Precuneus	L		5.57	-6	-62	34
MCC	R		3.91	2	-30	32
Precuneus	R		3.72	10	-54	32
Angular Gyrus	L	1597	6.54	-46	-62	28
Middle Temporal Gyrus	L	595	4.84	-54	-34	-6
Superior Frontal Gyrus	L	355	5.24	-14	52	28
Middle Frontal Gyrus	L	299	4.48	-26	18	54
Superior Frontal Gyrus	L		4.42	-12	30	54
Hippocampus	L	191	5.19	-32	-26	-14

Note: Combined sample. Only unique anatomical labels are reported for each cluster at one laterality. Whole-brain cluster-level FWE corrected (P < 0.001 uncorrected). FWE, Familywise error; L, left; MCC, midcingulate cortex; MNI, Montreal Neurological Institute; R, right.

Old vs. New effects: Old items yielded greater BOLD response than new items in six clusters. However, the two largest clusters, with peak voxels in precentral gyrus and cerebellum, indicate motor responses and are attributable to the fact that button-press sides for “Old” and “New” had not been randomized. Furthermore, a left middle frontal gyrus cluster; a left-hemispheric cluster comprised of inferior parietal lobule (IPL), angular gyrus and supramarginal gyrus (SMG); a left middle temporal gyrus cluster and one right-hemispheric cluster encompassing superior and middle occipital gyrus and angular gyrus were observed. For the reverse contrast (New>Old), the two corresponding contralateral clusters due to motor responses were found; a third and smaller cluster encompassed left rolandic operculum and left superior temporal gyrus.

Supplementary Table 2. Study Phase I, Type 1. Summary of significant clusters for the Old > New contrast.

Anatomical label	Laterality	Cluster size [k]	*T*-Value	Peak voxel MNI coordinates		
Precentral Gyrus	R	10355	17.3	38	-24	52
Postcentral Gyrus	R		13.58	50	-18	52
Rolandic Operculum	R		12.64	44	-20	18
Thalamus	R		7.2	14	-18	6
Putamen	R		7.11	30	-8	0
Caudate Nucleus	R		6.33	10	8	2
Cerebellum (IV-V)	L	7008	12.12	-16	-54	-16
Lingual Gyrus	L		10.92	-10	-70	-2
pMFC	R		7.05	8	-8	50
PCC	L		6.39	-8	-50	20
MCC	R		6.22	10	-16	50
MCC	L		6.0	-8	-44	38
Cuneus	L		5.7	-10	-66	28
Precuneus	L		5.66	-8	-66	38
Middle Frontal Gyrus	L	6283	8.15	-44	16	42
pMFC	L		6.31	-8	16	54
IFG (p. Triangularis)	L		6.3	-46	22	28
Superior Frontal Gyrus	L		6.26	-4	30	42
Inferior Parietal Lobule	L	2917	9.35	-38	-58	44
Angular Gyrus	L		9.18	-38	-62	46
Supramarginal Gyrus	L		4.05	-60	-48	34
Middle Temporal Gyrus	L	448	4.8	-64	-34	-12
Superior Occipital Gyrus	R	173	4.04	34	-74	42
Angular Gyrus	R		4.0	38	-70	42
Middle Occipital Gyrus	R		3.97	34	-72	38

Note: Combined sample. Only unique anatomical labels are reported for each cluster at one laterality. Whole-brain cluster-level FWE corrected ($P < 0.001$ uncorrected). FWE, Familywise error; IFG, inferior frontal gyrus, L, left; MCC, midcingulate cortex; MNI, Montreal Neurological Institute; PCC, posterior cingulate cortex; pMFC, posterior medial frontal cortex; R, right.

Supplementary Table 3. Study Phase I, Type 1. Summary of significant clusters for the New > Old contrast.

Anatomical label	Laterality	Cluster size [k]	*T*-Value	Peak voxel MNI coordinates		
Precentral Gyrus	L	2241	13.5	-42	-20	62
Postcentral Gyrus	L		4.38	-18	-42	66
Superior Parietal Lobule	L		4.35	-20	-38	64
Cerebellum (IV-V)	R	1291	9.52	16	-54	-16
Lingual Gyrus	R		7.9	20	-68	-6
Rolandic Operculum	L	423	7.23	-38	-22	18
Superior Temporal Gyrus	L		3.78	-54	-32	12

Note: Combined sample. Only unique anatomical labels are reported for each cluster at one laterality. Whole-brain cluster-level FWE corrected (P < 0.001 uncorrected). FWE, Familywise error; L, left; MNI, Montreal Neurological Institute; R, right.

Type 2 fMRI Analyses

LoP effects: Type 2 activations following retrieval of deeply processed items elicited significantly higher BOLD than shallowly processed items in right calcarine and lingual gyrus. There were no significant effects for the reverse contrast (P>0.05).

Supplementary Table 4. Study Phase I, Type 2. Summary of significant clusters for the Deep > Shallow contrast.

Anatomical label	Laterality	Cluster size [k]	*T-Value*	Peak voxel MNI coordinates		
Calcarine Gyrus	R	289	4.08	14	-86	8
Lingual Gyrus	R		3.9	16	-82	-6

Note: Combined sample. Only unique anatomical labels are reported for each cluster at one laterality. Whole-brain cluster-level FWE corrected (P < 0.001 uncorrected). FWE, Familywise error; L, left; MNI, Montreal Neurological Institute; R, right.

Old vs. New effects: There were no significant effects for the Old>New contrast (P>0.05). One cluster was significantly more active following newly presented items than following old items in right precentral and postcentral gyrus.

Supplementary Table 5. Study Phase I, Type 2. Summary of significant clusters for the New > Old contrast.

Anatomical label	Laterality	Cluster size [k]	*T-Value*	Peak voxel MNI coordinates		
Precentral Gyrus	R	841	6.07	38	-20	56
Postcentral Gyrus	R		3.6	38	-34	68

Note: Combined sample. Only unique anatomical labels are reported for each cluster at one laterality. Whole-brain cluster-level FWE corrected ($P < 0.001$ uncorrected). FWE, Familywise error; L, left; MNI, Montreal Neurological Institute; R, right.

Supplementary Table 6. Study Phase I, Type 2. Summary of significant clusters for the Ketamine > Placebo contrast (parametrically modulated by Confidence Rating).

Anatomical label	Laterality	Cluster size [k]	*T*-Value	Peak voxel MNI coordinates		
Lingual Gyrus	L	220	4.54	-20	-66	-8
Fusiform Gyrus	L		3.98	-28	-54	-8
Lingual Gyrus	R	194	4.22	22	-54	-8
Fusiform Gyrus	R		3.5	26	-66	-8
Calcarine Gyrus	L	191	4.79	-2	-72	18
Calcarine Gyrus	R		3.82	4	-62	12
Superior Parietal Lobule	R	137	3.7	40	-54	58

Note: Only unique anatomical labels are reported for each cluster at one laterality. Whole-brain cluster-level FWE corrected ($P < 0.001$ uncorrected). FWE, Familywise error; L, left; MNI, Montreal Neurological Institute; R, right.

Study Phase I: Discussion (Task Effects)

Significant LoP effects were demonstrated for both Type 1 and Type 2 sensitivity measures: Deeply processed word items were more frequently correctly categorized, and these categorizations were accompanied by higher metacognitive sensitivity than for shallowly processed items. Moreover, both Type 1 and Type 2 reaction times were shorter for deeply processed items. However, there was no significant LoP effect on metacognitive efficiency. On a brain functional level, accurate retrieval of deeply encoded items elicited a larger Type 1 BOLD than for shallowly encoded items in left-hemispheric clusters associated with the DMN (Andrews-Hanna, 2012), including MCC, precuneus, angular gyrus, middle temporal gyrus, superior frontal gyrus and hippocampus. Type 2 responses following correct retrieval of deeply encoded items were furthermore accompanied by activation of a right primary visual cortex cluster similar to the one observed in Report>Follow, suggesting that the use of word items may evoke vivid, imaginative processes that become stronger with increasing conscious level.

Accurate retrieval of old compared to new items yielded widespread activation in different cortical areas, most notably middle frontal gyrus with a peak in pMFC, inferior parietal, mid-temporal

and occipital activation. The reverse contrast elicited a larger BOLD in left rolandic operculum and superior temporal gyrus, brain regions prominently associated with language and speech (Brown *et al.*, 2009).

Study Phase II: Results (Task Effects)

During deep encoding trials, five clusters were significantly more active than during trials with shallow encoding: Superolateral prefrontal areas, left inferior frontal gyrus (IFG) and left middle temporal gyrus (first cluster); left angular gyrus (second cluster); left PCC (third cluster); IFG and middle temporal gyrus were furthermore identified as separate peaks of right-hemispheric fourth and fifth clusters.

Supplementary Table 7. Study Phase II. Summary of significant clusters for the Deep > Shallow contrast.

Anatomical label	Laterality	Cluster size [k]	*T*-Value	Peak voxel MNI coordinates		
Superior Frontal Gyrus	L	11534	13.45	-6	52	36
IFG (p. Triangularis)	L		11.58	-52	28	2
IFG (p. Orbitalis)	L		11.05	-40	22	-16
Superior Frontal Gyrus	R		9.34	6	56	24
Middle Temporal Gyrus	L		9.21	-56	-10	-16
Angular Gyrus	L	1345	9.38	-52	-64	24
PCC	L	866	6.68	-2	-50	26
IFG (p. Orbitalis)	R	552	6.85	52	32	-8
IFG (p. Triangularis)	R		4.62	54	28	10
Middle Temporal Gyrus	R	219	6.31	58	-4	-14
Superior Temporal Gyrus	R		3.9	50	-18	-8

Note: Combined sample. Only unique anatomical labels are reported for each cluster at one laterality. Whole-brain cluster-level FWE corrected ($P < 0.001$ uncorrected). FWE, Familywise error; IFG, inferior frontal gyrus; L, left; MNI, Montreal Neurological Institute; PCC, posterior cingulate cortex; R, right.

For the reverse contrast (Shallow>Deep), seven clusters were identified: First, an extended cluster comprised of right angular gyrus and IPL (bilaterally), with additional local maxima in the SPL (right) and precuneus (left). A second cluster encompassed peaks in the right superior frontal gyrus (bilaterally) and left-hemispheric local maxima in pMFC and middle frontal gyrus. In the remaining clusters, significant effects in bilateral middle frontal and precentral gyrus and right-hemispheric effects in superior orbital gyrus, IFG, inferior temporal gyrus and insula could be observed.

Supplementary Table 8. Study Phase II. Summary of significant clusters for the Shallow > Deep contrast.

Anatomical label	Laterality	Cluster size [k]	*T*-Value	Peak voxel MNI coordinates		
Angular Gyrus	R	12597	10.32	30	-58	42
Inferior Parietal Lobule	R		10.07	44	-40	52
Inferior Parietal Lobule	L		8.92	-44	-38	46
Superior Parietal Lobule	R		8.62	16	-64	52
Precuneus	L		7.77	-10	-70	50
Superior Frontal Gyrus	R	3009	8.64	24	2	60
Superior Frontal Gyrus	L		7.98	-22	4	60
pMFC	L		7.64	-10	2	54
Middle Frontal Gyrus	L		7.31	-24	8	52
Precentral Gyrus	L	1364	6.48	-56	4	38
IFG (p. Opercularis)	L		5.76	-52	6	24
Insula Lobe	L		5.09	-46	2	2
Middle Frontal Gyrus	R	1019	5.02	40	38	18
Superior Orbital Gyrus	R		4.07	22	54	-6
Precentral Gyrus	R	961	5.97	52	8	32
IFG (p. Opercularis)	R		4.49	50	8	14
Insula Lobe	R		3.85	36	14	8
Middle Frontal Gyrus	L	316	5.17	-44	40	24
Inferior Temporal Gyrus	R	272	6.09	56	-56	-8

Note: Combined sample. Only unique anatomical labels are reported for each cluster at one laterality. Whole-brain cluster-level FWE corrected ($P < 0.001$ uncorrected). FWE, Familywise error; IFG, inferior frontal gyrus; L, left; MNI, Montreal Neurological Institute; pMFC, posterior medial frontal cortex; R, right.

Study Phase II: Discussion (Task Effects)

Our results indicate that, overall, pleasantness-rating appears to be accompanied by activation of a distributed fronto-parietal network similar to the areas described by Honey, Honey, O'Loughlin *et al.* (2005), whereas syllable counting mainly relies on posterior parietal activation.

The pronounced behavioral LoP effects in Study Phase I were confirmed in Study Phase II. Type 1 and Type 2 sensitivity, and here also Type 2 efficiency were significantly higher for deeply processed items and the corresponding reaction times were shorter than for shallowly processed words.

Plots of data distribution

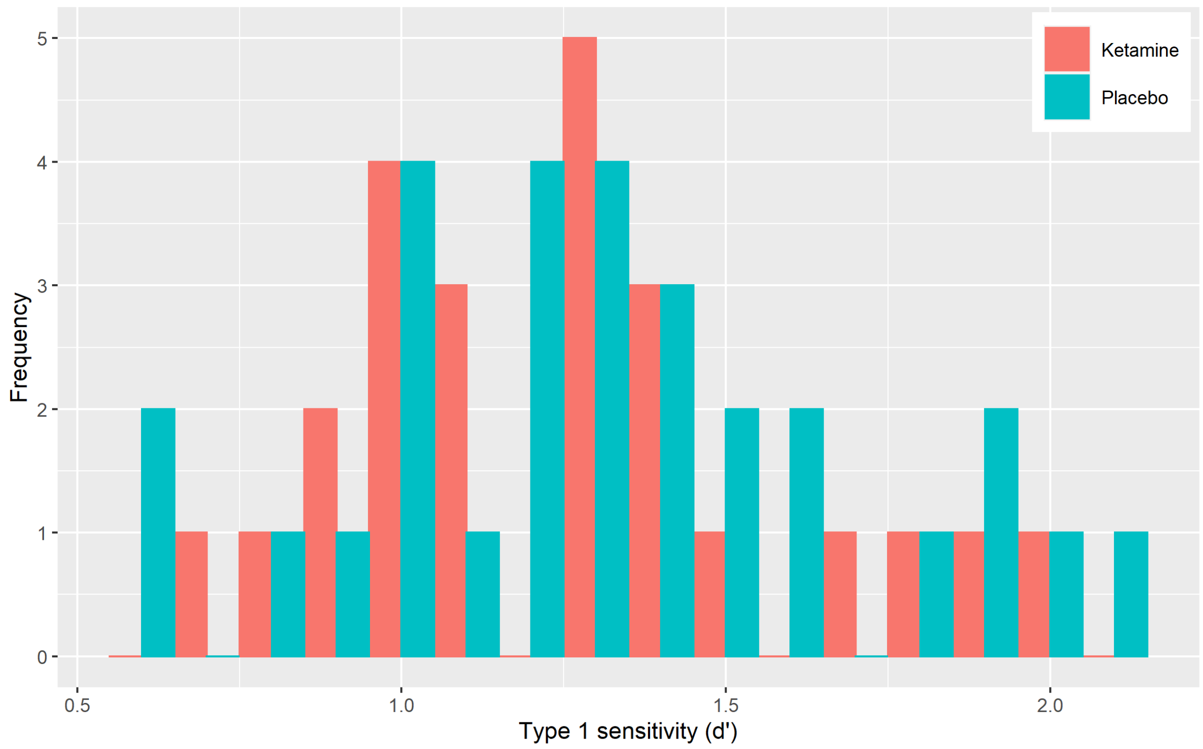

Supplementary Figure 1. Data distributions for Type 1 sensitivity (d’) in Study Phase I, by “Drug” (Ketamine/Placebo).

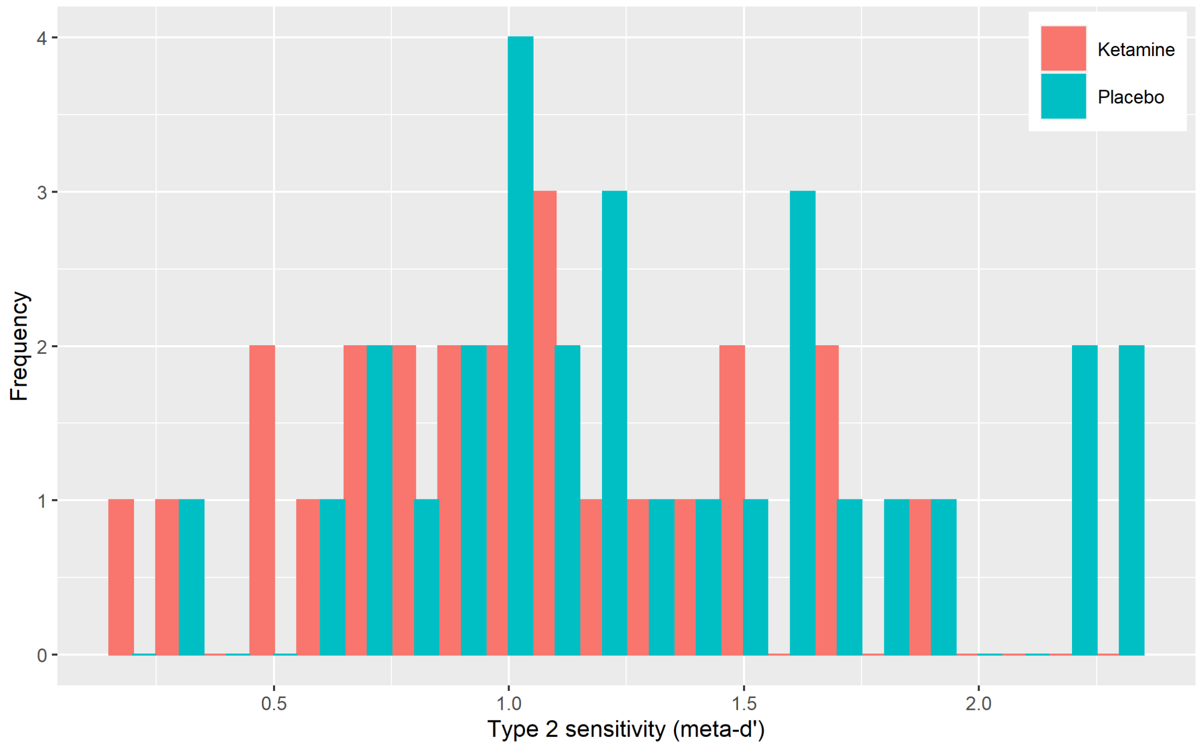

Supplementary Figure 2. Data distributions for Type 2 sensitivity (meta-d’) in Study Phase I, by “Drug” (Ketamine/Placebo).

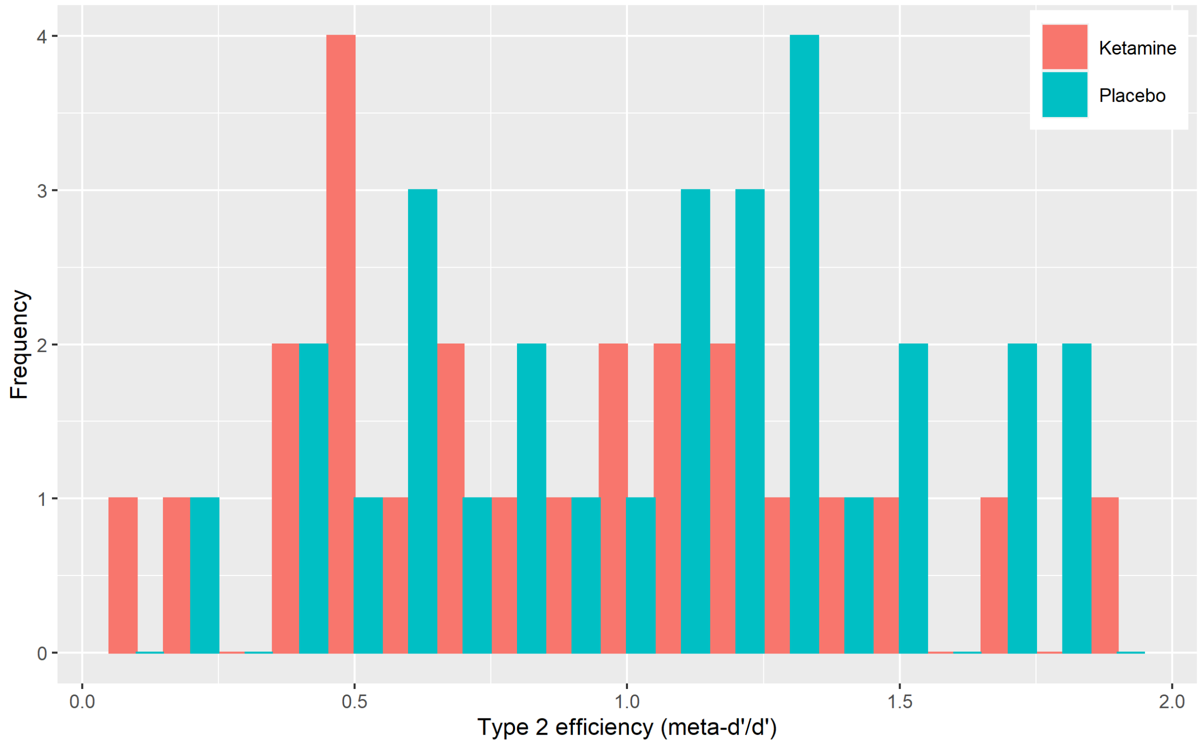

Supplementary Figure 3. Data distributions for Type 2 efficiency (meta-d'/d') in Study Phase I, by "Drug" (Ketamine/Placebo).

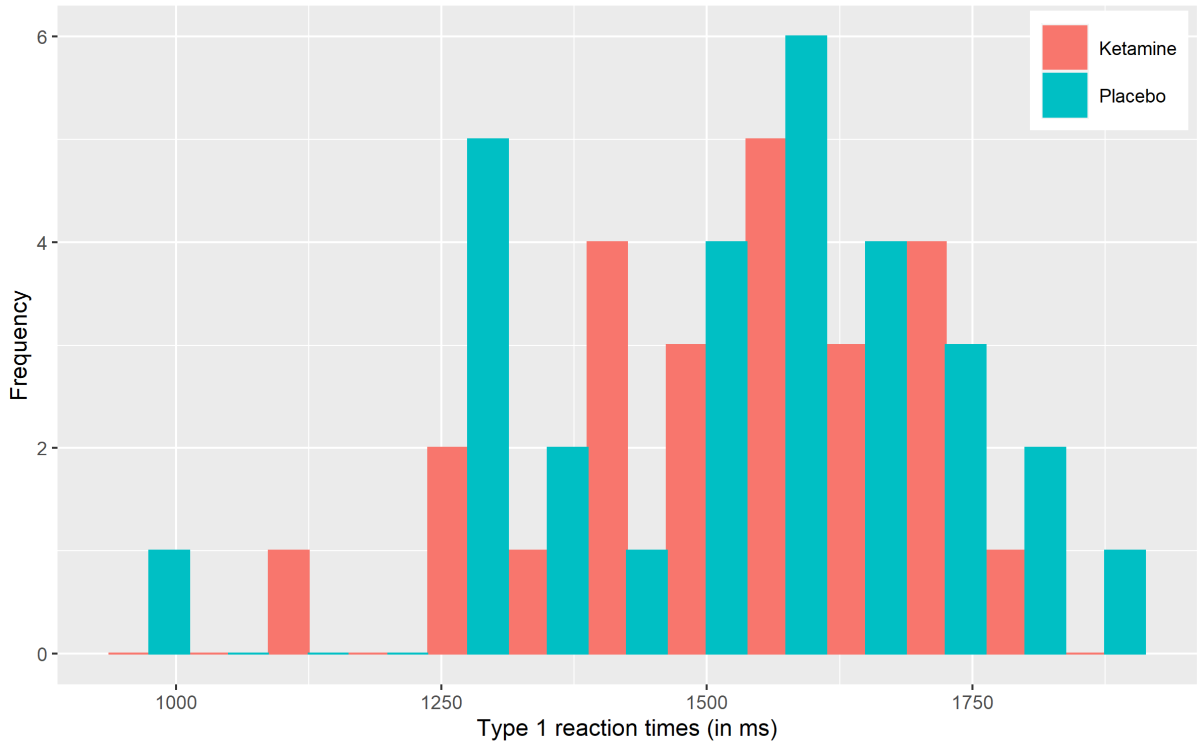

Supplementary Figure 4. Data distributions for Type 1 reaction times (in milliseconds) in Study Phase I, by "Drug" (Ketamine/Placebo).

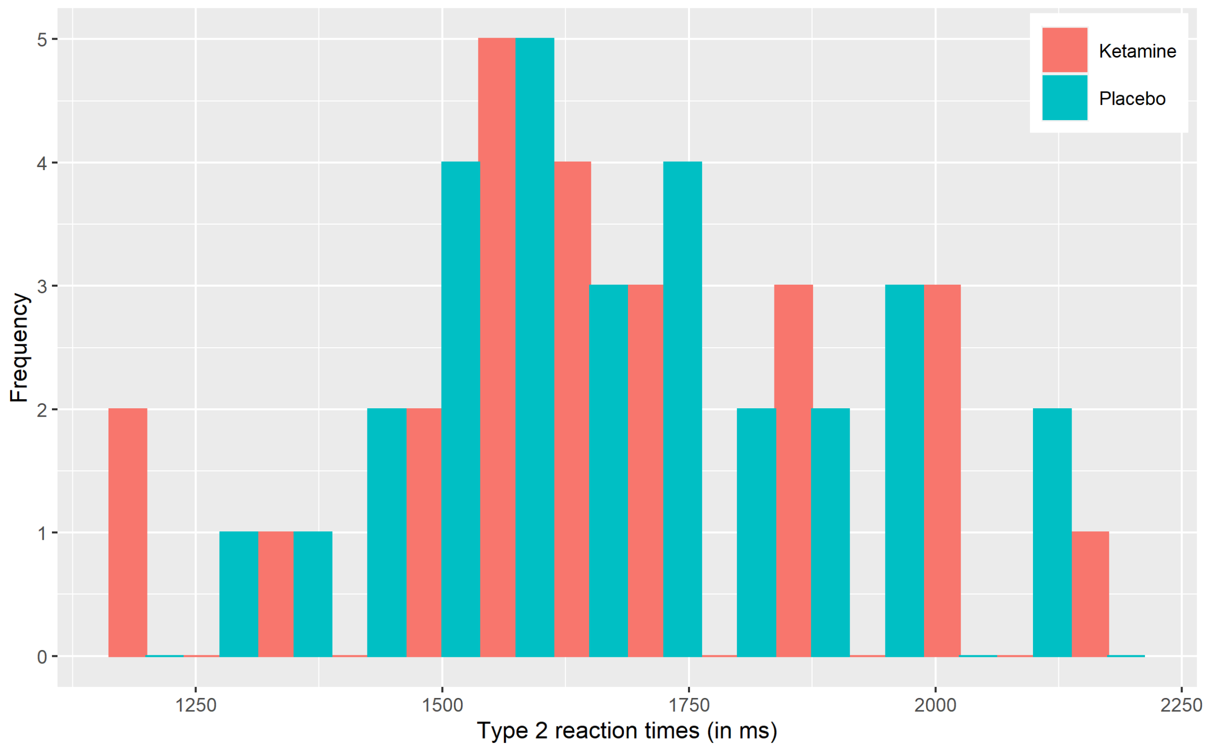

Supplementary Figure 5. Data distributions for Type 2 reaction times (in milliseconds) in Study Phase I, by ”Drug” (Ketamine/Placebo).

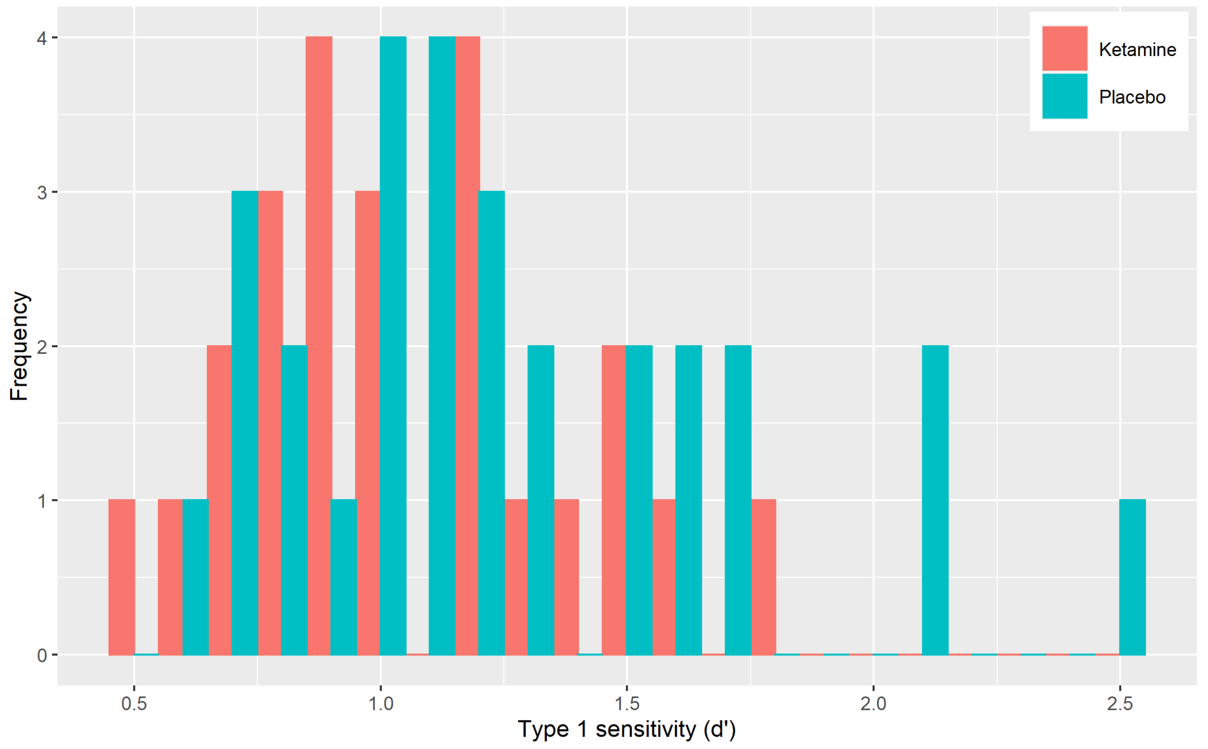

Supplementary Figure 6. Data distributions for Type 1 sensitivity (d’) in Study Phase II, by ”Drug” (Ketamine/Placebo).

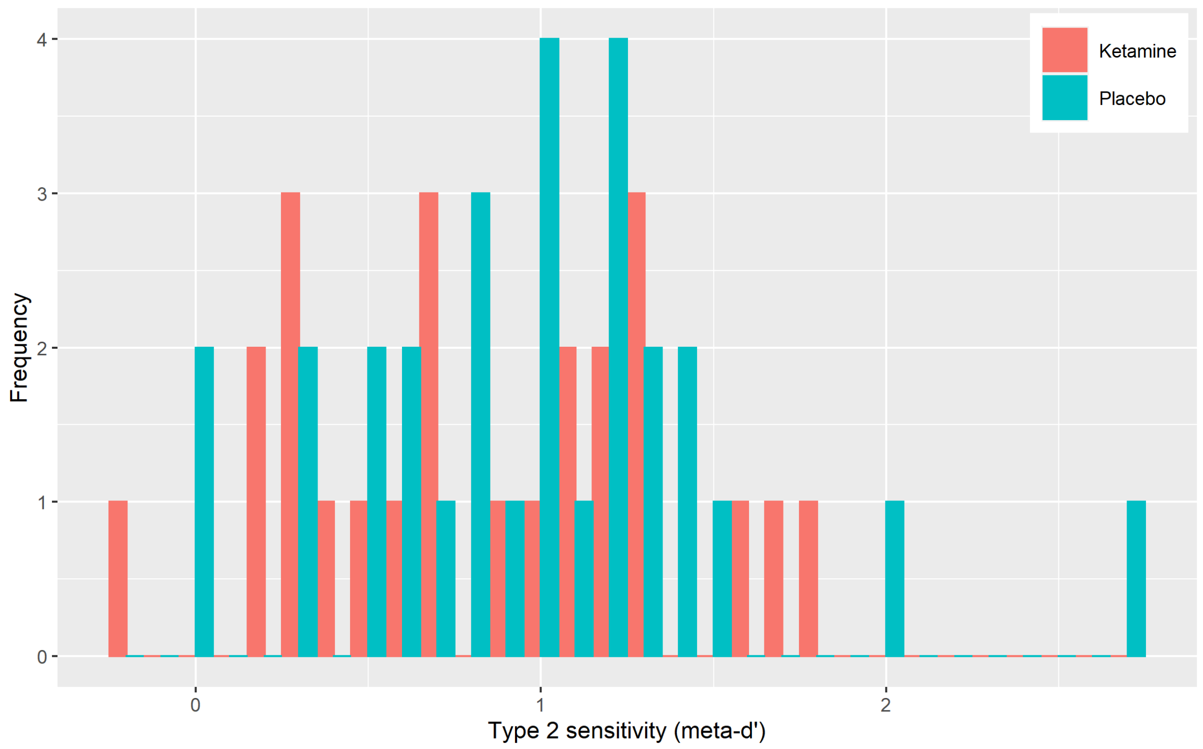

Supplementary Figure 7. Data distributions for Type 2 sensitivity (meta-d’) in Study Phase II, by ”Drug” (Ketamine/Placebo).

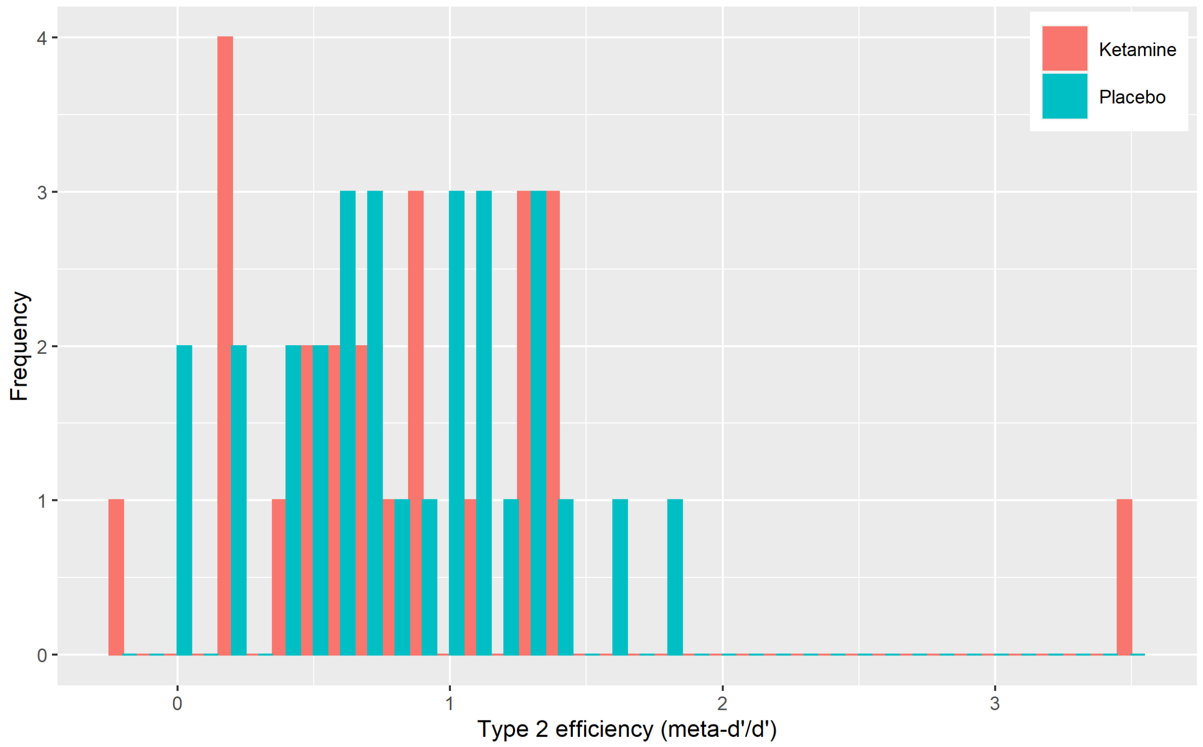

Supplementary Figure 8. Data distributions for Type 2 efficiency (meta-d’/d’) in Study Phase II, by ”Drug” (Ketamine/Placebo).

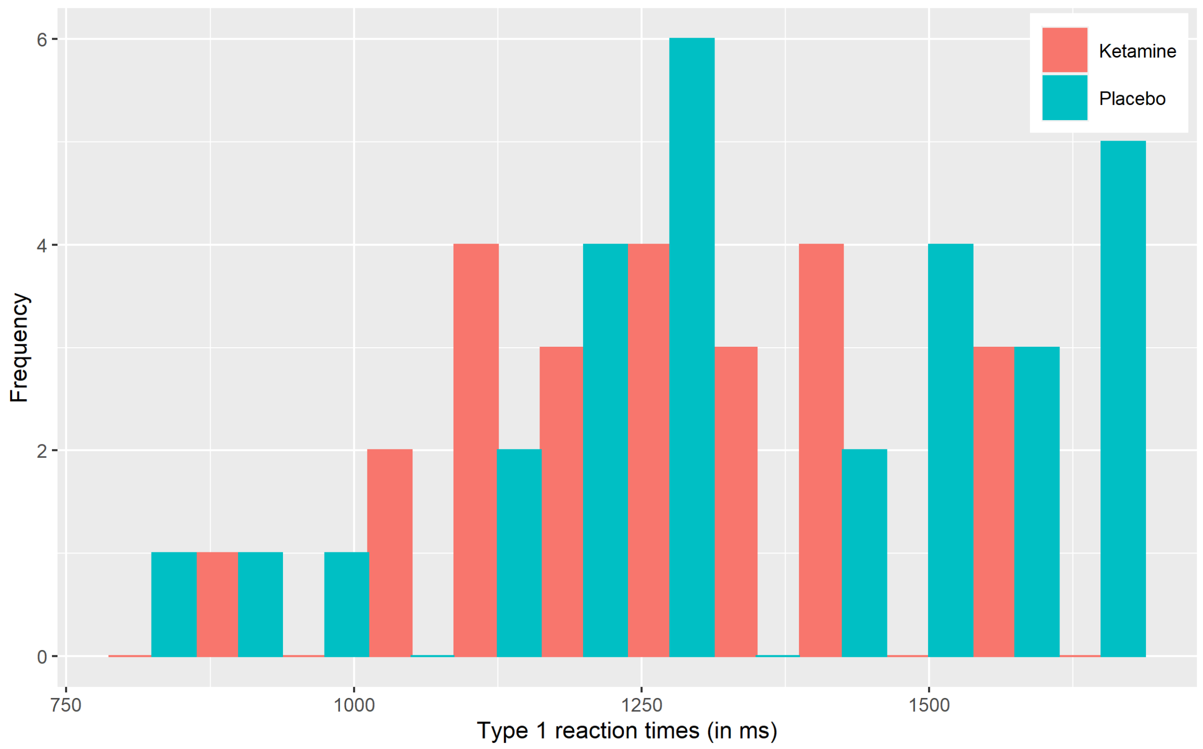

Supplementary Figure 9. Data distributions for Type 1 reaction times (in milliseconds) in Study Phase I, by ”Drug” (Ketamine/Placebo).

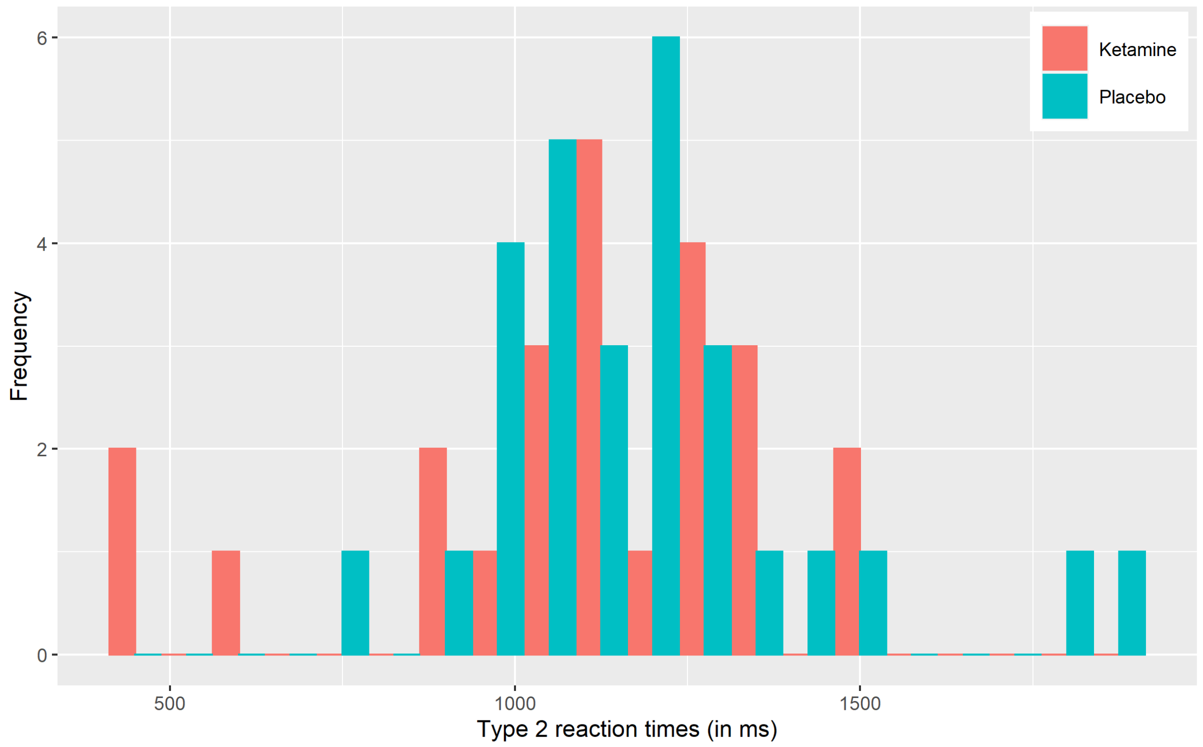

Supplementary Figure 10. Data distributions for Type 2 reaction times (in milliseconds) in Study Phase I, by ”Drug” (Ketamine/Placebo).

References

Andrews-Hanna, J. R. (2012). The brain's default network and its adaptive role in internal mentation. In *Neuroscientist*. https://doi.org/10.1177/1073858411403316

Brown, S., Laird, A. R., Pfordresher, P. Q., Thelen, S. M., Turkeltaub, P., & Liotti, M. (2009). The somatotopy of speech: phonation and articulation in the human motor cortex. *Brain and Cognition*, *70*(1), 31–41.

Honey, G. D., Honey, R. A. E., O'Loughlin, C., Sharar, S. R., Kumaran, D., Suckling, J., Menon, D. K., Sleator, C., Bullmore, E. T., & Fletcher, P. C. (2005). Ketamine disrupts frontal and hippocampal contribution to encoding and retrieval of episodic memory: An fMRI study. *Cerebral Cortex*, *15*(6), 749–759. https://doi.org/10.1093/cercor/bhh176
